# Safety and efficacy of cell therapies in pediatric heart disease: a systematic review and meta-analysis

**DOI:** 10.1186/s13287-020-01764-x

**Published:** 2020-07-08

**Authors:** John Martinez, Sarah Zoretic, Axel Moreira, Alvaro Moreira

**Affiliations:** 1 Department of Pediatrics, University of Texas Health San Antonio, San Antonio, TX 77229 USA; 2 Department of Pediatrics, Texas Children’s Hospital, Houston, TX 77030 USA; 3 Department of Pediatrics, UT Health San Antonio, 7703 Floyd Curl Drive, MC 7812, San Antonio, TX 78229 USA

**Keywords:** Regenerative medicine, Stem cells, Cardiology, Pediatric, Heart

## Abstract

**Background:**

Adult clinical trials have reported safety and the therapeutic potential of stem cells for cardiac disease. These observations have now translated to the pediatric arena. We conducted a meta-analysis to assess safety and efficacy of cell-based therapies in animal and human studies of pediatric heart disease.

**Methods and results:**

A literature search was conducted to examine the effects of cell-based therapies on: (i) safety and (ii) cardiac function. In total, 18 pre-clinical and 13 human studies were included. Pre-clinical: right ventricular dysfunction was the most common animal model (80%). Cardiac-derived (28%) and umbilical cord blood (24%) cells were delivered intravenously (36%) or intramyocardially (35%). Mortality was similar between cell-based and control groups (OR 0.94; 95% CI 0.05, 17.41). Cell-based treatments preserved ejection fraction by 6.9% (*p* < 0.01), while intramyocardial at a dose of 1–10 M cells/kg optimized ejection fraction. Clinical: single ventricle physiology was the most common cardiac disease (*n* = 9). Cardiac tissue was a frequent cell source, dosed from 3.0 × 10^5^ to 2.4 × 10^7^ cells/kg. A decrease in adverse events occurred in the cell-based cohort (OR 0.17, *p* < 0.01). Administration of cell-based therapies improved ejection fraction (MD 4.84; 95% CI 1.62, 8.07; *p* < 0.01).

**Conclusions:**

In this meta-analysis, cell-based therapies were safe and improved specific measures of cardiac function. Implications from this review may provide methodologic recommendations (source, dose, route, timing) for future clinical trials. Of note, many of the results described in this study pattern those seen in adult stem cell reviews and meta-analyses.

## Background

Less than one half of patients diagnosed with critical congenital heart disease (CCHD) will survive into adulthood [1]. CCHD represents a group of cardiac conditions requiring surgical intervention within the first year of life and affects ~ 1 in every 500 births in the USA annually [2, 3]. Although surgical advancements have prolonged survival, many patients will develop cardiac morbidities that will eventually necessitate heart transplantation. As pediatric donors are scarce, novel therapies are warranted to preserve cardiac function, reduce morbidity, and improve overall outcomes.

Literature in adults suggests regenerative therapies are safe and efficacious in patients with myocardial infarction, heart failure, and dilated cardiomyopathy [4–7]. These findings have prompted further investigation into the use of cell-based/derived therapies (CBT) in CCHD. Animal models mimicking CCHD have consistently demonstrated that stem/stromal cell therapies are safe and effective [8, 9]. Currently, early phase clinical trials utilizing regenerative cells for CCHD and other pediatric heart conditions are ongoing.

We conducted a systematic review and meta-analysis of pre-clinical and clinical studies to test the hypothesis that cell-based/derived therapies for congenital or acquired pediatric heart disease are safe and effective. In doing so, we hope to identify the variables that will have the greatest clinical impact and identify knowledge gaps that need to be addressed in future studies. Implications from this review may provide recommendations and evidence-based results for future human trials.

## Methods

The protocol for this review was submitted and registered with PROSPERO international database of systematic reviews and meta-analysis (Supplemental 1): registration number: CRD42019149559. Our methods adhere to the guidelines established by the Systematic Review Centre for Laboratory Animal Experimentation (SYRCLE) and are described in Supplemental 2.

### Literature search

Literature search was performed using PUBMED, Scopus, ScienceDirect, and Web of Science through September 3, 2019. Search terms included synonyms for “stem cell,” “regenerative therapy,” and “congenital heart disease” (full search terms available in Supplemental 3). Restrictions to the search were not placed on dates or languages. Reference lists of relevant reviews and included studies were examined.

After removal of duplicates, papers were screened by title and abstract and then evaluated for eligibility by full-text review performed by two independent investigators (JM, SZ). Differences of opinion in either phase were resolved by a third investigator (AM). Individual searches of reference lists of included studies were performed in an attempt to obtain additional studies for inclusion.

### Inclusion and exclusion criteria

Interventional and observational human studies assessing the safety and efficacy of CBT in congenital/pediatric heart disease were included. We defined cell-based therapies to include the following: mesenchymal stromal cells, embryonic cells, multipotent cells, inducible pluripotent cells, progenitor cells, hematopoietic cells, umbilical cord blood cells, c-kit+ cells, and cell derived as the secretome, exosomes, microRNA, microvesicles, and extracellular vesicles. Due to the paucity of human studies evaluating stem cell use in congenital heart disease, case reports were included. Studies including adult patients with a history of congenital heart disease (i.e., hypoplastic left heart syndrome (HLHS)) receiving CBT were included, whereas studies describing use of regenerative therapies in adult patients with other cardiac illnesses (i.e., myocardial infarctions) were excluded.

Pre-clinical studies were included if they reported the effect of CBT on validated in vivo animal models of congenital heart disease (e.g., RV overload). The uses of CBT were included regardless of dosage, timing, frequency, and source. Animal models of adult heart disease (myocardial infarction/ischemia) or healthy hearts were excluded.

### Primary endpoints

Our primary endpoints were safety and efficacy of cell-based/derived therapies in pre-clinical or clinical studies of both congenital and pediatric heart disease. Safety was defined as either mortality or the occurrence of adverse events (e.g., fever, rash, infection, hemodynamic instability, arrhythmia) associated with administration of CBT. Efficacy included measures of cardiac function: right ventricular ejection fraction, left ventricular ejection fraction (RVEF, LVEF), end diastolic or end systolic volumes (EDV, ESV), fractional area change (FAC), fractional shortening (FS), and tricuspid annular plane systolic excursion (TAPSE).

### Data extraction

Data was collected independently by two investigators (JM, SZ) and compared for accuracy. A third investigator (AM) assisted in resolution of difference of opinion. Extracted data included general study design (disease model, clinical trial phase, objective, sample size, inclusion/exclusion criteria), animal characteristics (species, age, gender, immune status), intervention characteristics (cell type, tissue source, dose, delivery, timing, frequency), and bibliographical information (author, year, funding, title, language, contact author email, journal) along with any measures related to our primary outcome as defined above. Study data was obtained from text, graphs, and plots. WebPlot digitizer was used to collect values from figures when not explicitly stated in the text.

For all studies where dosages were not reported in a cells/kg basis, the mean weight reported was used to calculate a mean dose of cells/kg. When an absolute cell count was provided, the mean weight (if present) was used to obtain a cells/kg dose. When two separate dosages were reported, mean dose/kg were obtained. When no weight was reported, absolute cell number was used. Such cases are denoted by an asterisk in Table 1.

**Table 1:** Overview of pre-clinical study details

Author (year)	Study design	Animal characteristics			Intervention characteristics		Outcome measures	
	Disease model	Species	Age	Immunosuppression	Cell type; source (origin)	Total dose (cells/kg), route, timing of delivery, frequency	Timing of assessment relative to injury	Ventricular function mode of assessment
Agarwal et al. [10]	RVHF	Crl:NIH-Foxn1rnu rats	5–6 weeks	Athymic	Cardiac progenitor (xenogeneic)	6.6 × 10^6^; intramyocardial; 2 weeks; once	Two weeks and 1 month	Echo
Albertario et al .[11]	RVHF	Landrace pigs	DNR	DNR	Thymic (xenogeneic)	5.0 × 10^5 a^; patch/graft; DNR; once	DNR	Echo, cardiac MRI
Borenstein et al.	RVHF	Ile de France rams	4 months	Dexamethasone	Skeletal muscle (autologous)	4.3 × 10^5^; intramyocardial; 3 h; once	Day 0 and day 60	Cath
Brizard et al. [12]	Cardiopulmonary bypass	Border-Leicester lamb	5–7 days	DNR	Umbilical cord blood (xenogeneic)	8.0 × 10^6^; intracoronary; intraoperative; once	DNR	Cath
Cao et al.	RVHF	New Zealand white rabbits	1 month	DNR	Bone marrow (autologous)	1.9 × 10^7^; intravenous; 3 months; once	Two weeks	Echo, Cath
Chery et al.	RVHF	RNU nude rats	DNR	DNR	Thymic (xenogeneic)	1.8 × 10^7^; cell sheet; 2 weeks; once	DNR	Echo
Davies et al .[13]	RVHF	Leicester sheep	10 days	Cyclosporine	Umbilical cord blood (xenogeneic)	7.0 × 10^5^; epicardial; 30 min; once	One month	Cath
Henning et al.	DCM	TO2 hamsters	1 month	DNR	Umbilical cord blood (xenogeneic)	5.3 × 10^7^; intramyocardial; 1 month; once	Monthly, 1 to 5 months	Echo
Lambert et al.	RVHF	Landrace pigs	2–3 months	Tacrolimus	Cardiac progenitor (xenogeneic)	4.7 × 10^5^; intramyocardial; 4 months; once	Four months and 7 months	Cath
Liu et al.	RVHF	Wistar lamb	DNR	DNR	Adipose (autologous)	5.0 × 10^7^; intravenous; 3 months; once	DNR	Echo, Cath
Nana-Leventaki et al .[14]	AI	Lewis rats	5–6 weeks	DNR	Cardiosphere derived (autologous)	1.7 × 10^6 b^; intracoronary; 10 days; once	One day, 10 days, 1 month	Echo
Schmuck et al.	RVHF	Sprague Dawley rats	5–6 weeks	DNR	Cardiac fibroblasts (xenogeneic)	2.0 × 10^6 a^; bioscaffold implanted into RV; 3 weeks; once	Baseline, 3 weeks and 6 weeks	Echo, Cath
Sugiura et al. (2016)	RVHF	Nude athymic rats	DNR	Athymic	Cardiac (xenogeneic)	2.0 × 10^5 a^; patch; DNR; once	Two and 4 months	Echo
Trac et al.	RVHF	Crl:NIH-Foxn1rnu rats	6 weeks	Athymic	Cardiac progenitor (xenogeneic)	3.0 × 10^6^; intramyocardial; 2 weeks; once	Weekly for 4 weeks	Echo
Umar et al. (2009)	RVHF	Wistar rat	8 weeks	DNR	Bone marrow-derived mesenchymal stem cells (autologous)	4.4 × 10^3^; intravenous; 2 weeks; once	Two weeks	Cath
Wehman et al. (2016)	RVHF	Yorkshire swine	2–3 weeks	Cyclosporine and methylprednisolone	Bone marrow (xenogeneic)	1.25 × 10^5^; intramyocardial; 30 min; once	Four months	Echo
Wehman et al. (2017)	RVHF	Yorkshire swine	2–3 weeks	Cyclosporine and methylprednisolone	Cardiac progenitor (xenogeneic)	1.25 × 10^5^; intramyocardial; morning of isolation; once	One day and 1 month	Echo
Yerebakan et al.	RVHF	Domestic sheep	4 months	DNR	Umbilical cord blood (autologous)	2.0 × 10^6^; intramyocardial; intraoperative; once	Six and 12 weeks	Echo, Cath

*RVHF* right ventricular heart failure, *DCM* dilated cardiomyopathy, *AI* autoimmune myocarditis, *RV* right ventricle, *DNR* did not report

^a^Absolute cell count

^b^Dose obtained based on additional study cited within paper

Additional cell-based/derived characteristics obtained included passage number, whether cells were self-isolated or purchased along with any positive surface markers noted in each study. When a study did not explicitly use a variable (e.g., EF), but measurements were provided such that it could be calculated (i.e., EDV, SV), these measurements were used to obtain variable of interest using the equation EF = SV/EDV [15].

### Data analysis

Meta-analysis was conducted using a random effects model. The estimated efficacy of regenerative therapies was determined using mean differences (MD) or standardized mean differences (SMD) with a 95% confidence interval (CI) [7]. SMD was used when studies reported the same outcome but reported in a different manner (e.g., MRI, echocardiogram, cardiac catheterization). For studies that described greater than two treatment groups, only the cell-based group and control were analyzed. Studies that did not include a control arm were described qualitatively.

Subgroup analysis was performed to assess for variability in safety and efficacy of CBT by route, dose, timing, source, and disease model. If multiple assessments of cardiac function were made in the article, they were regarded as separate experiments/datapoints. Cardiac variables were obtained at the time of induction of the cardiac model in animals and any subsequent measurements. In humans, baseline functional measures were compared to all timepoints after administration of CBT.

Statistical heterogeneity between studies was assessed via *I*^2^ with a value > 50% suggesting heterogeneity. Subgroup analysis was performed to evaluate for potential sources of heterogeneity [7]. Meta-analysis was conducted in R using the dmetar package [16]. All statistical tests were two-sided, and the difference was deemed significant for a *p* value < 0.05.

### Risk of bias

All studies were assessed for bias by two independent reviewers (JM, SZ). For animal studies, the SYRCLE risk of bias assessment tool was used [17], while ROBINS-I risk of bias was used for randomized/non-randomized control trials [18, 19]. Publication bias assessed through the use of funnel plots and Egger’s regression analysis. Funnel plots were visually assessed for asymmetry. For Egger’s test, *p* < 0.05 was considered significant to confirm the presence of a small study size.

## Results

**Fig. 1:**
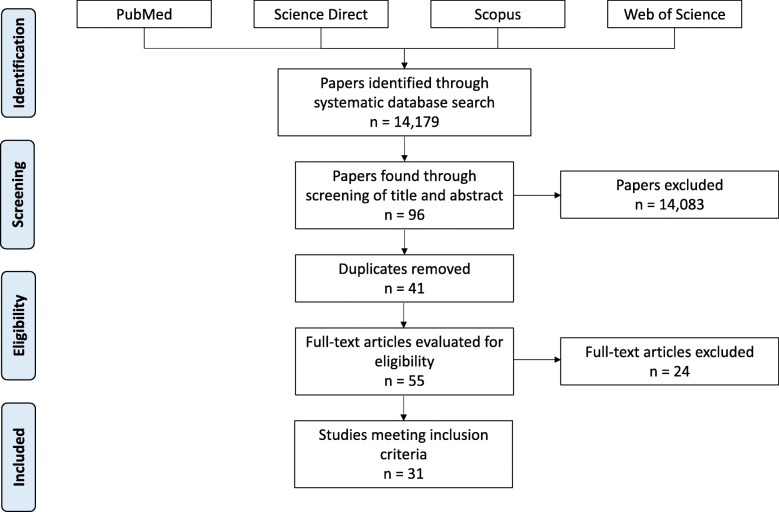
Flow diagram demonstrating study selection process

We identified 14,179 papers that met our broad inclusion criteria in four databases. Ninety-six studies were selected for abstract/summary review; 55 studies were chosen for full text/detailed review. After thorough evaluation, a total of 31 studies were included in this review (complete list provided in Supplemental 4), refer to Fig. 1 for details and Supplemental 5 for PRISMA checklist [20].

### Pre-clinical results

#### Study characteristics

Eighteen of the included studies were animal (*n* = 386). The model most commonly induced was right ventricular heart failure (RVHF) (*n* = 307, 80%), with the remaining studies modeling dilated cardiomyopathy (*n* = 45), autoimmune myocarditis (*n* = 22), and cardiopulmonary bypass (*n* = 12). There was a near-equal distribution of small (55%) and large animals (45%). Ninety-four percent of studies reported our primary outcomes (safety and/or efficacy). Study details are described in Table 1.

#### Intervention characteristics

While our search included both cell-based and cell-derived therapies, zero studies utilized cell-derived therapies. The majority of studies introduced non-autologous cell sources (*n* = 12, 67%). Tissue source of cells varied with cardiac and umbilical cord blood comprising most studies (*n* = 11, 61%). Intravenous (*n* = 140) and intramyocardial injection (*n* = 135) were the most frequent routes of cell delivery with a dose ranging from 4.4 × 10^3^ cells/kg to 5.3 × 10^7^ cells/kg. A summary of study characteristics is provided in Supplemental Table 1.

### Cell-based/derived therapies are safe in animals

#### Adverse events

Overall, there were no difference between CBT (16/409) and control groups (19/418) [Peto OR 0.89; 95% CI (0.43, 1.83); *p* = 0.74]. Specifically, cardiac events were most frequently observed (21/147); the CBT group yielded 11% while the control group had 18% [Peto OR 0.48; 95% CI (0.17, 1.33); *p* = 0.16]. The frequency of occurrence of overall and system-specific adverse events is outlined in Supplemental Table 3. Adverse event specifics are detailed in Supplemental Table 2.

#### Mortality

**Fig. 2:**
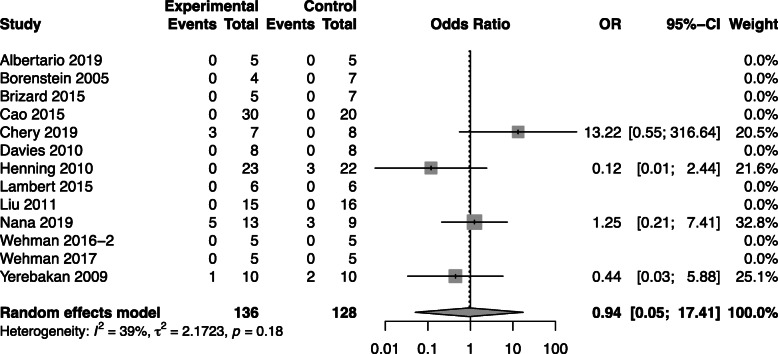
Effect size of regenerative cells on animal mortality. Forest plot demonstrating OR and 95% CI. Cell based, *n* = 136; Control, *n* = 128; *p* = 0.94. Rightward of the line of null effect favors cell based group, leftward favors control

A total of thirteen studies reported mortality outcomes (Fig. 2). No difference was noted in the risk of mortality between CBT and control groups [Peto OR 0.94; 95% CI (0.05, 17.41), *p* = 0.94].

### CBT preserves cardiac function in animals

#### Ejection fraction (EF)

**Fig. 3:**
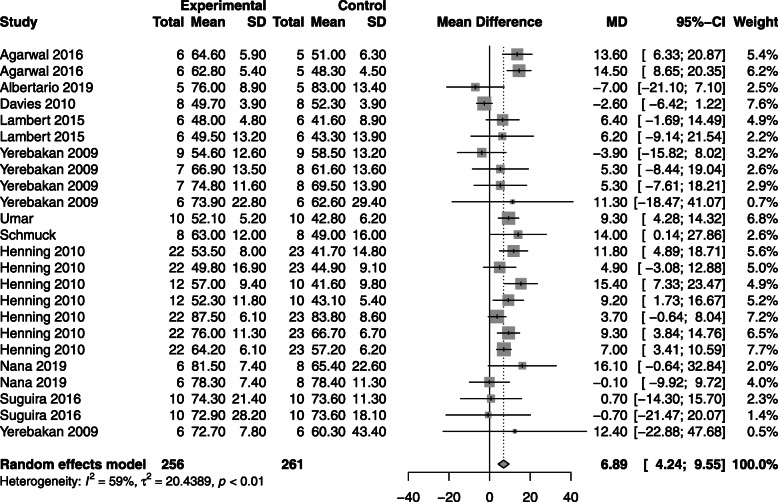
Effect size of regenerative cells on animal ejection fraction. Forest plot demonstrating MD and 95% CI. Cell based, *n* = 256; Control, *n* = 261; *p* < 0.00001. Rightward of the line of null effect favors cell based group, leftward favors control

Ten (56%) studies assessed either RVEF or LVEF. Animals treated with CBT had an increased EF compared to control [MD 6.9%; 95% CI (4.24, 9.55); *p* < 0.01], refer to Fig. 3 and Supplemental 6A-C.

In subgroup analysis, intramyocardial delivery of cells at a dose of 1–10 M cells/kg, administered between 1 week and 1 month after disease induction had the largest impact on EF (*p* < 0.01). Furthermore, cardiac-derived cells from a non-autologous source optimized EF in animals (*p* < 0.01). Specifics can be seen in Supplemental 7A-E. Bone marrow-derived mesenchymal stem cells (MSCs) demonstrated similar effects on EF to cardiac stem cells; however, there was only one study utilizing bone marrow MSCs.

#### Fractional shortening (FS)

**Fig. 4:**
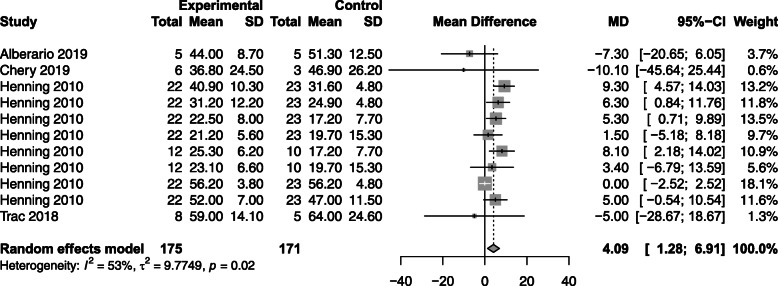
Effect size of regenerative cells on animal fractional shortening. Forest plot demonstrating MD and 95% CI. Cell based, *n* = 175; Control, *n* = 171; *p* = 0.004. Rightward of the line of null effect favors cell based group, leftward favors control

Of the four studies assessing FS (*n* = 83 animals), CBT demonstrated an improvement compared to controls [MD 4.09%; 95% CI (1.28, 6.91); *p* = 0.004 (Fig. 4)]. Subgroup analyses for FS paralleled the findings in EF for route; however, differed with regard to source, dose, and timing (Supplemental 8A-E).

#### Other cardiac outcomes

CBT treated trended towards preserving FAC (*p* = 0.05) (Supplemental 9A). No difference was appreciated with respect to EDV, ESV, and TAPSE between the groups (Supplemental 9B-D).

### Clinical outcomes

#### Study characteristics

Thirteen of the included studies were human (*n* = 215). HLHS or single-ventricle physiology was the most represented disease (*n* = 183), with dilated cardiomyopathy making up the remainder (*n* = 32). All studies reported safety and greater than 90% commented on efficacy of CBT (*n* = 206). Study details are described in Table 2.

**Table 2:** Overview of clinical study details

Author (year)	Study design	Human characteristics		Intervention characteristics		Outcome measures	
	Study type	Disease	Age	Cell type; source (origin)	Total dose (cells/kg); route; timing of delivery; frequency	Timing of assessment	Ventricular function mode of assessment
Burkhart et al. [21]	Case report	HLHS	4 months	Umbilical cord blood (autologous)	3.0 × 10^6^; intramyocardial; at time of stage II palliation; once	Baseline and 3 months	Echo
Burkhart et al. [22]	Phase I	HLHS	3–6 months	Umbilical cord blood (autologous)	3.0 × 10^6^; intramyocardial; during stage II palliation; once	Baseline, discharge, 1 month, 3 months, and 6 months	Echo
Eitoku et al. [23]	Phase I/II	SV	5–70 months	Cardiac (autologous)	3.0 × 10^5^; intracoronary; 1-month post-staged palliation; once	Baseline, 3 years	Cardiac MRI
Ishigami et al. (2014)	Phase I	HLHS	1.5 years	Cardiac (autologous)	3.0 × 10^5^; intracoronary; 1-month post-staged palliation; once	Baseline, 3 months, 6 months, 12 months, and 18 months	Echo, cardiac MRI
Ishigami et al. [24]	Phase II	SV	1.2–4.2 years	Cardiac (autologous)	3.0 × 10^5^; intracoronary; 1-month post-staged palliation; once	Baseline, 3 months	Cardiac MRI
Pincott et al. [25]	Phase I/II	DCM	1–16 years	Bone marrow (autologous)	5.4 × 10^7b^; intracoronary; within 1 h of harvest; once	Baseline, 6 moths	Cardiac MRI, Cath
Qureshi et al. [26]	Case report	HLHS	25 years	Bone marrow (autologous)	2.0 × 10^6^; intracoronary; within 3 h of harvest; once	Baseline, 1 month, 3 months, 6 months	Echo
Rivas et al. [27]	Case report	DCM	3–4 months	Bone marrow (autologous)	8.35 × 10^6a^; intracoronary; 2 h post-isolation; once	Baseline, 1 week, 4 weeks, 8 weeks, and 17 weeks	Echo
Rupp et al. [28]	Case report	HLHS	11 months	Bone marrow (autologous)	DNR; intracoronary; day of harvest; once	Baseline, 3 months	Echo, cardiac MRI
Rupp et al. [29]	Phase I/II	DCM	4 months-16 years	Bone marrow (autologous)	DNR; intracoronary; day of harvest; once	Baseline, 2–3 months, 24–52 months	DNR
Sano et al. [30]	Phase I	SV	8 months-3.1 years	Cardiac (autologous)	3.0 × 10^5^; intracoronary; intraoperative (stage II or III); once	Baseline, 1 year, 2 years post-palliation	Echo
Tarui et al. [31]	Phase I	HLHS	1.5 years	Cardiac (autologous)	3.0 × 10^5^; intracoronary; 1-month post-staged palliation; once	Baseline, 36 months	Cardiac MRI
Zschirnt et al. [32]	Case report	DCM	DNR	Bone marrow (autologous)	2.4 × 10^7^; intracoronary; DNR; once	Baseline, 4 months	Cardiac MRI

*HLHS* hypoplastic left heart syndrome, *SV* single ventricle, *DCM* dilated cardiomyopathy, *DNR* did not report

^a^Absolute cell count

^b^Dose obtained based on additional study cited within paper

#### Intervention characteristics

All human studies isolated autologous cells. Cardiac tissue (*n* = 170, 79%) with intracoronary infusion (*n* = 204, 95%) was the most common tissue source and form of delivery, respectively. The studies administered CBT intraoperatively or greater than 1 month after isolation of cells at a dose of 3.0 × 10^5^ cells/kg to 5.4 × 10^7^ cells/kg. A summary of study characteristics is provided in Supplemental Table 4.

### Cell-based/derived therapies are safe in humans

#### Adverse events

Patients treated with CBT had less adverse events compared to controls [44/492 vs. 98/606, Peto OR 0.17; 95% CI (0.09, 0.30); *p* < 0.01]. Cardiac and respiratory events were most frequently reported and both favored CBT [Peto OR 0.11; 95% CI (0.05, 0.23); *p* < 0.01] and [Peto OR 0.16; 95% CI (0.03, 0.95); *p* < 0.05], respectively. No difference was observed in GI, hematologic, infectious, or systemic events. The frequency of occurrence of overall and system-specific adverse events is outlined in Supplemental Table 5. Adverse event specifics are detailed in Supplemental Table 2.

#### Mortality

One human study reported events of mortality, which demonstrated a 7.3% death rate for CBT versus 15% for the control group (*p* > 0.05) [30].

### CBT preserves cardiac function in humans

#### Ejection fraction (EF)

**Fig. 5:**
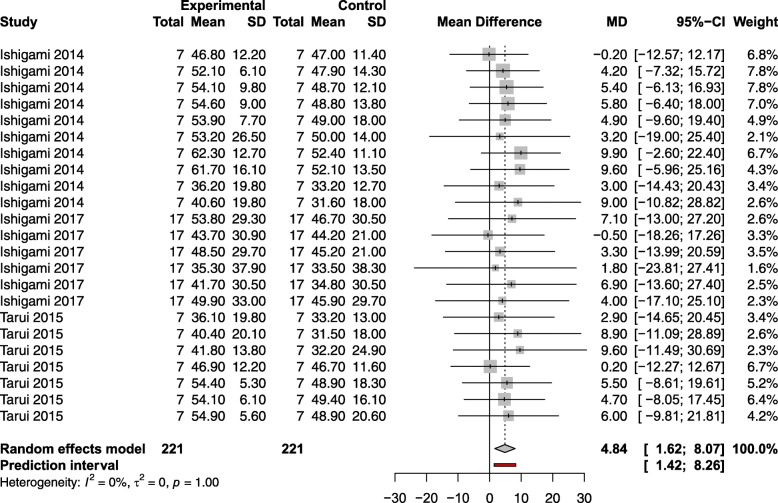
Effect size of regenerative cells on human ejection fraction. Forest plot demonstrating MD and 95% CI. Cell based, *n* = 221; Control, *n* = 221; *p* = 0.003. Rightward of the line of null effect favors cell based group, leftward favors control

CBT preserved EF compared to controls [MD 4.84; 95% CI (1.62, 8.07); *p* < 0.05], refer to Fig. 5. While twelve of thirteen studies assessed EF, only three of those studies underwent meta-analysis due to lack of a suitable control arm in the remaining [24, 31, 33]. One study did not report efficacy [29]. Given the paucity of studies assessing EF, subgroup analysis in the human studies was not performed.

#### Narrative findings of EF

Nine studies underwent qualitative analysis due to lack of comparison groups or absence of specified efficacy outcomes [21–23, 25–28, 30, 32]. Overall, the majority of CBT-treated patients demonstrated a trend towards increased EF compared to baseline. Most notably, Sano et al. observed an 8.4% improvement in EF vs. 1.6% in control following stage II palliation and a 7.9% improvement vs. − 1.1% at stage III palliation. Of note, Sano was excluded from meta-analysis given outcomes reported in terms of delta EF rather than absolute change. Rupp and Rivas reported an improvement in EF by 22% from baseline at the 3 and 4-month follow-up, respectively [27, 28]. Pincott et al. noted no difference in EF compared to baseline [25]. Burkhart et al. and Eitoku both observed improvement in RVEF in their respective studies [21, 23].

Notably, Zschirnt reports a substantial improvement in LVEF of 24% from baseline [32]. Results from Burkart et al. 2019 indicate that intramyocardial injection of umbilical cord blood-derived cells at the time of stage II palliation preserves baseline RVEF [22]. Lastly, Qureshi et al showed an increase in EF from 34 to 38% by cardiac magnetic resonance imaging at the 6-month follow-up in their case report [26].

#### Other cardiac outcomes

Zero human studies assessed the effects of CBT on FS. FAC and EDV were analyzed in three studies (Supplemental 10A and 10B, respectively). Three studies provided data for ESV analysis (Supplemental 10C). Three studies mentioned TAPSE, but none could undergo analysis due to the absence of a comparison arm. In sum, no significant differences were observed in FAC, EDV, or ESV when comparing CBT to control (Supplementals 10A–C).

### Risk of bias

No animal study fulfilled all ten criteria for low risk of bias (Supplemental 11). One study [12] met eight of the criteria for a low risk of bias and four [10, 11, 14, 34] studies met seven of the criteria. All studies were deemed to be at low risk of bias for similar groups at baseline, complete data, and selective reporting. One study was at high risk for other biases [13]. Visually apparent asymmetry was present in the funnel plot of fractional shortening while minimal asymmetry was present in the funnel plot of ejection fraction, thus denoting a lack of publication bias (Supplemental Figs. 13–14).

The ROBINS-I risk of bias assessment tool was employed for human studies (Supplemental 12, 13, and 14). One study fulfilled all five criteria for low risk of bias [25]. All studies performed their intended interventions without deviations and reported complete data with a low risk of bias [21–33]. Ten studies described randomization procedures with a high risk of bias [21–23, 26–32], while only one study demonstrated concerns of bias for selective reporting [28].

## Discussion

Our review performed a meta-analysis of cell-based therapies used in the treatment of animal models of pediatric heart disease and in pediatric patients with congenital and acquired cardiac dysfunction.

Our outcomes of interest included safety and efficacy. Safety was stratified into mortality and the occurrence of adverse events, which were assessed by system-specific prevalence. Cell-based therapies proved to be safe in comparison to placebo in both animal and human studies. Efficacy was defined by various measures of cardiac function. Of those, cell therapies preserved ejection fraction in animals and humans. To further understand variables that may modulate effect size on ejection fraction, subgroup analysis was performed in pre-clinical studies based on delivery route, timing of administration, tissue source, and dose.

To date, pediatric clinical trials are evaluating the safety of cell-based interventions for hypoplastic left heart syndrome. Standard of care for this condition entails a three-staged surgical approach over the first 2 years of life. However, a subset of individuals develops right ventricular failure, as this chamber is not anatomically or physiologically adept to maintain systemic circulation. The current goal of cell-based therapies is to bridge the patient during surgical inter-stages by maintaining right ventricular function. Therefore, the use of cell therapies in pediatric heart disease is being investigated as an adjunct to surgery versus surgery alone.

Timing of delivery appeared to have the greatest effect size on ejection fraction in pre-clinical studies. Cell therapies administered 1 week to 1 month after disease model induction preserved EF by 10.6%. The impact of timing of cellular delivery on efficacy is well recognized. Hu et al. described a time-dependent therapeutic effect in a rat model of myocardial infarction (MI). Rats receiving intramyocardial injection 1 week after induction of disease model demonstrated the greatest preservation in EF compared to those receiving cell therapies at initial induction or at a later time point [35]. Crisostomo et al. observed comparable results in a larger swine model of MI. Swine receiving intracoronary cardiac porcine cells at 2 h post induction of disease model demonstrated greater decline in EF in comparison to those that received cells 7 days post induction [36]. These results have translated to human studies. A meta-analysis performed by Xu et al. of 34 randomized control trials (RCT) revealed that administration of the autologous bone marrow cells 3–7 days post percutaneous coronary intervention optimized efficacy [37]. Timing of delivery appears to play a role in the efficacy of cell-based therapies. Immediate administration at onset of disease model appears sub-optimal. Nitkin et al. described a critical “inflection” point in the disease course where therapeutic effects are maximal [38]. Future clinical studies of cellular therapies for pediatric heart disease should prioritize elucidating the optimal timing of cellular administration.

The studies included herein transplant the stem cells in a variety of administrative routes, including intracoronary, intramyocardial, intravenous, graft/patch/sheet, and epicardial. Intramyocardial delivery of stem cells demonstrated the most favorable effect on EF in pre-clinical studies with a MD of 7.35%. Although intravenous delivery of CBT yielded a comparable effect on EF in animal studies (9.3%), only one study evaluated this route, and therefore, results should be taken guardedly. A meta-analysis published by Kanelidis et al. demonstrated how the route of delivery modulates the efficacy of mesenchymal stem cell therapy for MI in pre-clinical and clinical studies. While transendocardial stem cell injection yielded the greatest improvement in infarct size and EF for both pre-clinical and clinical studies, the study also illustrated beneficial effects of intramyocardial delivery on EF in pre-clinical studies. Of note, intramyocardial delivery of stem cells has not been studied in trials focusing on acute MI as this route requires open-heart surgery and deviates from the current standard of treatment which entails cardiac cathetherization [7]. Brunskill et al. also investigated the route of delivery and found that the intramyocardial delivery of stem cells optimizes the effects of trials investigating acute MI and ischemic heart disease [39]. Altogether, these findings suggest that the route of cell therapy delivery may affect cardiac efficacy.

Cell therapies of cardiac origin demonstrated a 9.2% preservation of EF in animal models. Cardiac stem cells have historically been an attractive therapeutic option due to their high angiogenic potential, balanced paracrine profile, and ability to differentiate into myocardial lineages when compared to other cell types [40, 41]. A meta-analysis performed by Zwetsloot et al. including 1970 animals receiving cardiac cells demonstrated an improved ejection fraction by 10.7%. When stratifying by type of cardiac cell (cardiosphere derived, cardiosphere, c-kit+, sca-1+), variable improvements of 12.9%, 11.8%, 10.7, and 8.3% were observed [41]. Translating these findings into human clinical trials remains challenging. For instance, a recently published phase I/II double-blinded RCT in patients with ST-segment elevation MI revealed no significant difference between patients receiving cardiac stem cells and placebo [42]. Moreover, there exists considerable heterogeneity in tissue source being utilized in pediatric clinical trials. In this review, bone marrow-derived MSCs demonstrated similar effects on EF to cardiac stem cells (9.3%), yet these results should be interpreted with caution as only one study included the use of bone marrow MSCs. Of the included pediatric clinical studies in this review, 38% are utilizing CBT of cardiac origin whereas the remainder provided umbilical cord blood and bone marrow MSCs. Future trials should examine the tissue source most likely to provide cardiac benefit.

This review revealed that a dose of 1–10 M cells/kg modulated EF by 8.4%. It is unsurprising that the efficacy of cell therapies appears to be dose-dependent. This observation has been reported in both pre-clinical studies and human clinical trials of acute myocardial infarction. Tang et al. describes a rat model of MI where five different escalating doses of cardiac stem cells were tested (0.3 × 10^6^, 0.75 × 10^6^,1.5 × 10^6^, 3.0 × 10^6^, 6.0 × 10^6^). The lowest dose had no effect on LV function; doses ranging 0.75–3.0 × 10^6^ provided a similar cardiac benefit. Interestingly, post procedure mortality increased with 6.0 × 10^6^ cells. These results suggest an optimal dose required for efficacy in addition to a dosage plateau where no additional benefits are observed [43]. Spoel and colleagues note similar findings in a meta-analysis of 52 large animal models utilizing cardiac stem cells for treatment of acute MI. Transplantation of doses greater than 10^7^ cells had the greatest impact on improvement in LVEF [44]. Xu and co-authors performed a meta-analysis including 40 RCT, assessing 1927 patients, receiving bone marrow-derived cells in the treatment of acute myocardial infarction. There appears to be a dose-dependent effect in efficacy. No differences were observed in patients receiving ≤ 10^7^ cells or 10^9^ cells [37]. These findings provide evidence that an optimal dose will also be observed in pediatric patients. Of the 13 human studies included in this review, 30% are utilizing doses between 1 and 10 M cells/kg with high variability in dosage between remaining studies.

As noted previously, studies on cell therapies in animal models with MI have shown favorable effects on cardiac function. While these studies prompted investigation for the use of stem cells in pediatric heart disease, one must be cautious when comparing degrees of efficacy from adult to pediatric studies. The pathophysiology of heart failure in the setting of myocardial infarction differs from that which causes heart failure in children. Moreover, heart failure in pediatric heart disease is largely attributed to right ventricular dysfunction as opposed to left ventricular dysfunction in adults. Ischemic heart failure is most frequently due to LV systolic dysfunction with the leading cause due to loss of functional myocardium secondary to ischemic disease [45], whereas heart failure in children can be attributed to several mechanisms including alterations to pressure or volume load, valvular abnormalities, electromechanical dysynchrony, coronary anomalies, and myocardial fibrosis [46]. While previous studies on cell therapies in adults can provide significant insight, it is unsuitable to make a direct comparison of cell-based therapies and their potential effects in two varied populations.

This review evaluated the safety of administration of cell-based therapies in pre-clinical models of pediatric heart disease and in children with congenital or acquired heart disease. Cell-based therapies are generally regarded as safe for administration. In this review, no significant differences were observed in animal mortality or occurrence of adverse events. Only one of thirteen human studies explicitly commented on mortality. An important finding in human studies was that less cardiac and respiratory events were observed in the cell-based group. Demonstrating safety after CBT was paralleled in a meta-analysis done by Thompson et al. of MSCs delivered across a multitude of diseases. Compared to controls, there was no increased risk of death, infusion toxicity, infection, thrombotic/embolic events, or malignancy. However, there was an acutely increased risk of fever associated with administration of cell products compared to control [47]. In another review, Lalu et al. report no significant differences in the occurrence of adverse events in the cell group vs control group [4].

### Limitations

Although the results of our meta-analysis and subsequent subgroup analysis are promising, they should be interpreted with caution. It is conceivable that the observed optimal route of delivery, dose, timing, and tissue source may not truly reflect ideal therapeutic parameters. Moreover, they may be a mere reflection of the parameters most often used by investigators in the field. Future studies will need to individually investigate and optimize each variable to achieve maximal therapeutic benefits. An additional limitation of this study arises from the early stage of human clinical trials for congenital and acquired pediatric heart disease. The simple lack of data created a challenge in completion of a robust meta-analysis for human studies. As more trials are completed, a future meta-analysis needs to be performed to assess the reproducibility of our reported results. Currently, there are six ongoing clinical trials (Supplemental Table 6).

A large degree of heterogeneity was detected in both the pre-clinical and clinical studies included in this review. Recently, the Transnational Alliance for Regenerative Therapies in Cardiovascular Syndromes (TACTICS) identified areas of focus in cell-based therapies for cardiovascular disease. Moreover, the methodologic rigor of translational research was pinpointed as a main area of concern, wherein the experts proposed the following themes as ways to improve translational success: internal validity, standardization of protocols (cell source, dose, timing, etc.), and multicenter animal studies [48]. This lack of standardization has led to a high degree of variability in the efficacy of regenerative therapies for pediatric heart disease. In doing so, perhaps we will begin to see more consistently reproducible positive human results.

## Conclusion

Cell-based therapies are safe in both animal models of pediatric heart disease and in human clinical trials of congenital and acquired cardiac disease. These cells appear to serve a role in the preservation of specific measures of cardiac function. Their efficacy appears modulated by the route and timing of delivery, dose, and tissue source. Implications from this review may provide methodologic recommendations for current and future clinical studies.

## Supplementary information

**Additional file 1 Supplemental 1.** PROSPERO registration **Supplemental 2.** SYRCLE criteria for animal intervention studies. **Supplemental 3.** Database search terms. **Supplemental 4.** List of included studies. **Supplemental 5.** PRISMA Checklist. **Supplemental 6.** Effect size of regenerative cell on animal ejection fraction. Forest plots demonstrating MD and 95% CI for A) Left ventricular ejection fraction; cell-based *n*=172; control=177; *p* <0.0001. B) Right ventricular ejection fraction; cell-based *n*=84; control n=84; *p*=0.02. C) Disease model; cell-based *n*= 256; control *n*=261; RVHF, *p*=0.01; DCM, *p*<0.0001. **Supplemental 7.** Subgroup analysis of regenerative cell effect size on animal ejection fraction. Forest plots demonstrating MD and 95% CI for A) Route of delivery, *p*<0.00001 for intramyocardial injection. B) Dose, *p*<0.00001 for 1-10 M. C) Tissue Source, *p*<0.0001 for cardiac; *p*=0.0003 for bone marrow. D) Timing of delivery, *p*<0.0001 for 1 week–1 month. E) autologous vs. non-autologous sources, *p*<0.0001 (non-autologous). Cell-based n=256; Control n=261. **Supplemental 8.** Subgroup analysis of regenerative cell effect size on animal fractional shortening. Forest plots demonstrating MD and 95% CI for A) Route of delivery, *p*=0.001 for intramyocardial. B) Dose, *p*=0.001 for >10 M cells/kg. C) Tissue Source, *p*=0 .001 for umbilical cord blood. D) Timing of delivery, *p*=0.001 for >1 month. E) Disease model, *p*=0.001 for dilated cardiomyopathy. Cell-based *n*=175; Control *n*=171. **Supplemental 9.** Effect size of regenerative cell on additional measures of animal cardiac function. Forest plots demonstrating MD and 95% CI for A) Fractional area change, *p*=0.05; cell-based *n*= 33; control *n*=30. B) End diastolic volume, *p*=0.48; cell-based *n*=67; control *n*=61. C) End systolic volume, *p*=0.60; cell-based *n*=72; control *n*=66. D) Tricuspid annular plane systolic excursion, *p*=0.55; cell-based *n*=33; control *n*=58. **Supplemental 10.** Effect size of regenerative cell on additional measures of human cardiac function. Forest plots demonstrating MD and 95% CI for A) Fractional area change, *p*-0.19; cell-based *n*=62; control *n*=62. B) End diastolic volume, *p*=0.52; cell-based *n*=110; control *n*=110. C) End systolic volume, *p*=0.96; cell-based *n*=110; control *n*=110. **Supplemental 11.** SYRCLE risk of bias for animal studies. **Supplemental 12.** ROBINS-I risk of bias for human studies. **Supplemental 13.** Funnel plot diagram for animal ejection fraction. **Supplemental 14.** Funnel plot diagram for animal fractional shortening. **Supplemental Table 1.** Animal study intervention characteristics. **Supplemental Table 2.** Adverse events by systems. **Supplemental Table 3.** Animal adverse events. **Supplemental Table 4.** Human study intervention characteristics. **Supplemental Table 5.** Human adverse events. **Supplemental Table 6.** Clinical trials, ongoing.

## Data Availability

The data underlying this article are available in the article and in its online supplementary material.

## Abbreviations

CBT: Cell-based/derived therapies
CCHD: Critical congenital heart disease
CI: Confidence interval
EDV: End diastolic volume
ESV: End systolic volume
EF: Ejection fraction
FAC: Fractional area change
FS: Fractional shortening
HLHS: Hypoplastic left heart syndrome
LVEF: Left ventricular ejection fraction
MD: Mean difference
MRI: Magnetic resonance imaging
OR: Odds ratio
PROSPERO: International database of systematic reviews and meta-analysis
ROBINS: Risk of bias in non-randomized studies of interventions
RV: Right ventricle
RVEF: Right ventricular ejection fraction
SYRCLE: Systematic Review Centre for Laboratory Animal Experimentation
TAPSE: Tricuspid annular plane systolic excursion
