# Supplementary material for: Safety and efficacy of cell therapies in pediatric heart disease: a systematic review and meta-analysis
Source: Stem Cell Res Ther. 2020 Jul 8;11:272. doi: 10.1186/s13287-020-01764-x (PMC7341627; doi:10.1186/s13287-020-01764-x)
Supplement: Supplementary file 1 — Additional file 1 Supplemental 1. PROSPERO registration Supplemental 2. SYRCLE criteria for animal intervention studies. Supplemental 3. Database search terms. Supplemental 4. List of included studies. Supplemental 5. PRISMA Checklist. Supplemental 6. Effect size of regenerative cell on animal ejection fraction. Forest plots demonstrating MD and 95% CI for A) Left ventricular ejection fraction; cell-based n=172; control=177; p <0.0001. B) Right ventricular ejection fraction; cell-based n=84; control n=84; p=0.02. C) Disease model; cell-based n= 256; control n=261; RVHF, p=0.01; DCM, p<0.0001. Supplemental 7. Subgroup analysis of regenerative cell effect size on animal ejection fraction. Forest plots demonstrating MD and 95% CI for A) Route of delivery, p<0.00001 for intramyocardial injection. B) Dose, p<0.00001 for 1-10 M. C) Tissue Source, p<0.0001 for cardiac; p=0.0003 for bone marrow. D) Timing of delivery, p<0.0001 for 1 week–1 month. E) autologous vs. non-autologous sources, p<0.0001 (non-autologous). Cell-based n=256; Control n=261. Supplemental 8. Subgroup analysis of regenerative cell effect size on animal fractional shortening. Forest plots demonstrating MD and 95% CI for A) Route of delivery, p=0.001 for intramyocardial. B) Dose, p=0.001 for >10 M cells/kg. C) Tissue Source, p=0 .001 for umbilical cord blood. D) Timing of delivery, p=0.001 for >1 month. E) Disease model, p=0.001 for dilated cardiomyopathy. Cell-based n=175; Control n=171. Supplemental 9. Effect size of regenerative cell on additional measures of animal cardiac function. Forest plots demonstrating MD and 95% CI for A) Fractional area change, p=0.05; cell-based n= 33; control n=30. B) End diastolic volume, p=0.48; cell-based n=67; control n=61. C) End systolic volume, p=0.60; cell-based n=72; control n=66. D) Tricuspid annular plane systolic excursion, p=0.55; cell-based n=33; control n=58. Supplemental 10. Effect size of regenerative cell on additional measures of human cardiac function. Fo [file 13287_2020_1764_MOESM1_ESM.pdf]

SUPPLEMENTAL MATERIAL

Supplemental 1. PROSPERO registration.

PROSPERO
International prospective register of systematic reviews

NHS
National Institute for Health Research

Animal review

1. * Review title.

Give the working title of the review. This must be in English. The title should have the interventions or exposures being reviewed and the associated health or social problems.

Safety and efficacy of cell-based/derived therapies in congenital heart disease; a systematic review and meta analysis of pre-clinical and clinical studies

2. Original language title.

For reviews in languages other than English, this field should be used to enter the title in the language of the review. This will be displayed together with the English language title.

English

3. * Anticipated or actual start date.

Give the date when the systematic review commenced, or is expected to commence.

27/08/2019

4. * Anticipated completion date.

Give the date by which the review is expected to be completed.

01/01/2020

5. * Stage of review at time of this submission.

Indicate the stage of progress of the review by ticking the relevant Started and Completed boxes. Additional information may be added in the free text box provided.
Please note: Reviews that have progressed beyond the point of completing data extraction at the time of initial registration are not eligible for inclusion in PROSPERO. Should evidence of incorrect status and/or completion date being supplied at the time of submission come to light, the content of the PROSPERO record will be removed leaving only the title and named contact details and a statement that inaccuracies in the stage of the review date had been identified.
This field should be updated when any amendments are made to a published record and on completion and publication of the review.

The review has not yet started: No

Review stage	Started	Completed
Preliminary searches	No	Yes
Piloting of the study selection process	No	Yes
Formal screening of search results against eligibility criteria	No	Yes
Data extraction	No	Yes
Risk of bias (quality) assessment	Yes	No
Data analysis	Yes	No

Provide any other relevant information about the stage of the review here (e.g. Funded proposal, protocol not yet finalised).

6. * Named contact.

The named contact acts as the guarantor for the accuracy of the information presented in the register record.

Alvaro Moreira

Email salutation (e.g. "Dr Smith" or "Joanne") for correspondence:

Dr Moreira

7. * Named contact email.

Enter the electronic mail address of the named contact.

MoreiraA@uthscsa.edu

8. * Named contact address.

Enter the full postal address for the named contact.

UT Health San AntonioDepartment of Pediatrics, Division of Neonatology7703 Floyd Curl Drive MC 7812San

Antonio, TX, USA 78229

9. Named contact phone number

Enter the telephone number for the named contact, including international dialling code.

210-567-5226

10. * Organisational affiliation of the review.

Full title of the organisational affiliations for this review and website address if available. This field may be completed as ‘none’ if the review is not affiliated to any organisation.

UT Health San Antonio

Organisation web address:

11. * Review team members and their organisational affiliations.

Give the personal details and the organisational affiliations of each member of the review team. Affiliation refers to groups or organisations to which review team members belong. **NOTE: email and country are**

now mandatory fields for each person.

Dr John Martinez. UT Health Pediatrics
Dr Sarah Zoretic. UT Health Pediatrics
Dr Alvaro Moreira. UT Health San Antonio, Department of Pediatrics, Division of Neonatology

12. * Funding sources/sponsors.

Give details of the individuals, organisations, groups or other legal entities who take responsibility for initiating, managing, sponsoring and/or financing the review. Any unique identification numbers assigned to the review by the individuals or bodies listed should be included.

Parker B. Francis

Grant number(s)

13. * Conflicts of interest.

List any conditions that could lead to actual or perceived undue influence on judgements concerning the main topic investigated in the review.

None

14. Collaborators.

Give the name, affiliation and role of any individuals or organisations who are working on the review but who are not listed as review team members.

15. * Review question.

Give details of the question to be addressed by the review, clearly and precisely.

Are cell-based/derived therapies both safe and efficacious in clinical trials involving congenital heart disease?

Context and rationale

Provide a brief description of the context and rationale of the review, including information on the relevance of your review for human health (max 250 words).

Preclinical studies have established that regenerative therapies show promise as primary/adjunctive therapies for congenital heart disease (CHD). Animal models have demonstrated that regenerative cells are safe and effective. As these therapies have now translated to clinical trials in pediatric CHD, it is imperative to summarize the current findings and identify knowledge gaps that still remain in order optimize translational success. Therefore, the purpose of this systematic review and meta-analysis is twofold: (i) assess the safety, and (ii) efficacy of cell-based/derived therapies in animal models of congenital heart disease.

16. * Searches.

Give details of the sources to be searched, and any restrictions (e.g. language or publication period). The full search strategy is not required, but may be supplied as a link or attachment.

MEDLINE via PubMed, Scopus, ScienceDirect, Web of Science, Reference lists of included studies, Reference lists of relevant reviews Search dates: no restriction on timeline of search results (initial year-08/26/19)Restrictions on language: no restrictions Publication: no publication date restrictions Will

searches be re-run prior to final analysis? : yes Will unpublished studies be sought? : no

17. URL to search strategy.

Give a link to the search strategy or an example of a search strategy for a specific database if available (including the keywords that will be used in the search strategies).

MEDLINE PubMed: ((((((((((((((((((((((regenerative[MeSH Terms]) OR regenerative) OR stem cell) OR stromal cell) OR mesenchymal) OR embryonic) OR pluripotent) OR multipotent) OR inducible pluripotent) OR progenitor) OR hematopoietic) OR umbilical cord) OR cord blood) OR c-kit) OR secretome) OR microRNA) OR exosome) OR microvesicle) OR microparticle) OR extracellular vessicle))) AND ((((((((((((((truncus arteriosus) OR transposition of great arteries) OR transposition of great vessels) OR tricuspid atresia) OR hypoplastic right heart syndrome) OR hypoplastic left heart syndrome) OR single ventricle) OR tetralogy of fallot) OR total anomalous pulmonary venous return) OR pulmonary atresia) OR coarctation of aorta) OR interrupted aortic arch) OR doublet outlet right ventricle) OR single inlet ventricle)

Alternatively, upload your search strategy to CRD in pdf format. Please note that by doing so you are consenting to the file being made publicly accessible.

Do not make this file publicly available until the review is complete

18. * Human disease modelled.

Give a short description of the disease, condition or healthcare domain being modelled.

Congenital heart disease

19. * Animals/population.

Give summary criteria for the animals being studied by the review, e.g. species, sex, details of disease model. Please include details of both inclusion and exclusion criteria.

Inclusion criteria:

Human: Children (newborn-18 years) with congenital heart disease to include adults with history of congenital heart disease receiving cell-based/derived therapies

Animal models of congenital heart disease

Exclusion criteria:

Human: Children without congenital heart disease, Adults without congenital heart disease receiving cell-based/derived therapies, Adult models of heart disease

Animal: Animal models without congenital heart disease

20. * Intervention(s), exposure(s).

Give full and clear descriptions of the nature of the interventions or the exposures to be reviewed (e.g. dosage, timing, frequency). Please include details of both inclusion and exclusion criteria.

Inclusion criteria:

The following will be used for both human and animal studies. Regenerative cell-based/derived therapy used to treat congenital heart disease. Regenerative cell therapies will be defined as: mesenchymal, embryonic, multipotent, inducible pluripotent cells, progenitor, hematopoietic, umbilical cord, cord blood, c-kit+, secretome, exosome, microRNA, microvesicles, extracellular vesicles.

Exclusion criteria:

The following will be used for both human and animal studies. Non cell-based/derived therapies used to treat congenital heart disease

21. * Comparator(s)/control.

Where relevant, give details of the type(s) of control interventions against which the experimental condition(s) will be compared (e.g. another intervention or a non-exposed control group). Please include details of both inclusion and exclusion criteria.

Inclusion criteria:

Human: Placebo. Children with congenital heart disease who did not receive cell based therapies.

Animal: Animals in experimental models not subject to cell-based/derived therapies for the treatment of congenital heart disease (placebo and sham).

Exclusion criteria:

Human: Children without congenital heart diseases

Animal: Animals not modeling congenital heart disease

22. * Study designs to be included.

Give details of the study designs eligible for inclusion in the review. If there are no restrictions on the types of study design eligible for inclusion, or certain study types are excluded, this should be stated. Please include details of both inclusion and exclusion criteria.

Inclusion criteria:

Clinical trials, cohort, case reports

Exclusion criteria:

Articles not assessing outcomes of interest

23. Other selection criteria or limitations applied.

Give details of any other inclusion and exclusion criteria, e.g. publication types (reviews, conference abstracts), publication date, or language restrictions.

Review articles, book chapters, abstracts will be excluded. No restrictions placed based on publication date or language.

24. * Outcome measure(s).

Give detail of the outcome measures to be considered for inclusion in the review. Please include details of both inclusion and exclusion criteria.

Inclusion criteria:

The following outcome measures will be used for both human and animal studies

Cardiac function, as measured by:

-Right/Left ejection fraction-End diastolic volume-End systolic volume-Tricuspid annular plane systolic excursion-Fractional area change-Fractional shortening Safety: -Mortality -Adverse events with administration (fever, rash, infection, hemodynamic instability, arrhythmia, etc)

Exclusion criteria:

Animal or human studies not assessing safety or efficacy (as defined above) after cell-based/derived.

25. N/A.

This question does not apply to systematic reviews of animal studies for human health submissions.

26. * Study selection and data extraction.

Procedure for study selection

Give the procedure for selecting studies for the review, including the screening phases (title and/or title-abstract and/or full-text), the number of researchers involved, and how discrepancies will be resolved.

Study selection: a) Two investigators (J. Martinez & S. Zoretic) will independently screen all the abstracts/full texts for the inclusion criteria. b) Differences of opinion in either phase that cannot be resolved by discussion will be resolved by consulting a third investigator (A. Moreira).

Prioritise the exclusion criteria

Multiple exclusion criteria may apply to an abstract/paper, which can cause discrepancies between reviewers in the reason for exclusion recorded. To avoid this, it is helpful to prioritize the exclusion criteria (e.g. 1) not an animal study; 2) not a myocardial infarction model, etc.) and record the highest ranking applicable criterion as the reason for exclusion. Please sort the exclusion criteria defined in questions 19 to 24. If applicable, do so for each screening phase.

1) in-vitro studies2) studies not including cell-based/derived therapies 3) Human studies not including congenital heart disease models 4) Animal models without congenital heart disease5) Animal or human studies not assessing safety or efficacy (as defined above) after cell-based/derived. 6) Adults without congenital heart disease receiving cell- based/derived therapies 7) Adult models of heart disease 8) Review articles, book chapters, abstracts

Methods for data extraction

Describe methods for data extraction, including the number of reviewers performing data extraction, extraction of data from text and/or graphs, whether and how authors of eligible studies will be contacted to provide missing or additional data, etc.

Data extraction: Study design, methodology, patient demographics, clinical diagnoses, cell characteristics (source, dose, frequency and delivery), cardiac imaging parameters, laboratory values, publication details (author, year, funding, etc), follow up data a) Two investigators (J. Martinez & S. Zoretic) will independently screen all the abstracts/full texts for the inclusion criteria. b) Differences of opinion in either phase that cannot be resolved by discussion will be resolved by consulting a third investigator (A. Moreira). Data will be extracted from text, tables and figures (webplot digitizer). For missing data, will contact authors. Data will be recorded via excel spreadsheet.

Data to be extracted: study design

Specify the data to be extracted related to characteristics of the study design, e.g. controlled versus cross-over, number of experimental groups, etc.

Humans: Number of children in experimental +/- control group, number of experimental groups, phase of clinical trial, cell-based/derived therapies parameters (dose, frequency, route, etc), cardiac assessments (echo, MRI, CT, biomarkers), time points for data collection

Animals: Number of animals in experimental +/- control group, number of experimental groups, cell-based/derived therapies parameters (dose, frequency, route, etc), cardiac assessments (echo, MRI, CT, biomarkers), time points for data collection

Data to be extracted: animal model

Specify the data to be extracted related to characteristics of the animal model, e.g. species, sex of the animals, etc.

Number of animals in experimental and control groups, power calculation reported, method(s) to induce congenital heart disease, animal species/strain, age, gender, weight and immune status.

Data to be extracted: intervention of interest

Specify the data to be extracted related to characteristics of the intervention of interest, e.g. dose, timing, etc.

Cell type, tissue source, dose, mode of delivery, frequency, timing, passage number

Data to be extracted: primary outcome(s)

Define the primary outcome measure(s). For each outcome measure, specify in which format data will be extracted, including the eligible units of measurement, and data type (continuous/dichotomous). A description of any other manipulation or transformation of the extracted data that is planned may be included.

The following outcomes will be assessed in both animal and human studies

Safety:

-Mortality (dichotomous) -Adverse events with administration (fever, rash, infection, hemodynamic instability, arrhythmia, etc) (dichotomous)

Assessment of cardiac function as measured by:

-Right/Left ejection fraction (%, continuous)-End diastolic volume (mL, continuous)-End systolic volume (mL, continuous) -Tricuspid annular plane systolic excursion (mm, cm, continuous) -Fractional area change (%, continuous) -Fractional shortening (%, continuous)

Data to be extracted: secondary outcome(s)

Define the secondary outcome measure(s). For each outcome measure, specify in which format data will be extracted, including the eligible units of measurement, and data type (continuous/dichotomous). A description of any other manipulation or transformation of the extracted data that is planned may be included.

n/a

Data to be extracted: other

Specify any other data or study characteristics to be extracted, e.g. bibliographical details, such as author, year and language.

Author, year, funding, title, language, contact author email, journal

27. * Risk of bias and/or quality assessment.

State whether and how risk of bias and/or study quality will be assessed. Assessment tools specific for pre-clinical animal studies include SYRCLE's risk of bias tool and the CAMARADES checklist for study quality

No risk of bias and/or quality assessment planned

No

By use of SYRCLE's risk of bias tool

Yes

By use of SYRCLE's risk of bias tool adapted as follows:

No

By use of the CAMARADES checklist for study quality

No

By use of the CAMARADES checklist for study quality, adapted as follows:

No

Other criteria, namely

Yes

Animal: SYRCLE Risk of bias

Human non randomized: Robins-I

Human randomized: Cochrane Risk of bias

Method for risk of bias and/or quality assessment

Give the procedure for the risk of bias and/or quality assessment, including the number of reviewers involved, their contribution, and how discrepancies will be resolved.

Two separate reviewers will assess risk of bias for each study. Discrepancies will be resolved by senior author.

28. * Strategy for data synthesis.

Planned approach

For each outcome measure, specify whether a quantitative or narrative synthesis is planned and how this decision will be made.

Quantitative synthesis will be preferred method for reporting information, however if 4 studies are assessing a particular outcome we will conduct a narrative explanation as, too few studies will be available to conduct meta-analysis.

If a meta-analysis is planned, please specify the following:

Effect measure

For each outcome measure, specify the effect measure to be used (e.g. mean difference, odds ratio etc.).

Animal studies: standardized mean difference

Human studies: odds ratio

Effect models

For each outcome measure, specify the statistical model of analysis (e.g. random-effects or fixed-effect model).

Random-effects model

Heterogeneity

Specify the statistical methods to assess heterogeneity (e.g. I^2, Q). For further guidance please refer to the introduction and practical guide to pre-clinical meta-analysis.

I^2

Other

Specify other details of the meta-analysis methodology (e.g. correction for multiple testing, correction for multiple use of control group).

n/a

29. * Analysis of subgroups or subsets.

Subgroup analyses

Give any planned exploration of subgroups or subsets within the review. ‘None planned’ is a valid response

if no subgroup analyses are planned.

Study design: experimental and control groups, congenital heart disease model, measures of safety

measures of function, outcome time

Animal models: species, strain, age, gender

Cell-based/derived therapy source: dose, delivery, timing, frequency, transplant method (allogeneic,

xenogeneic, autologous)

Sensitivity

For each outcome measure, specify any sensitivity analyses you propose to perform.

If high heterogeneity is observed (70%), subgroup analyses will be conducted

Publication bias

Specify whether an assessment of publication bias is planned. If applicable, specify the method for assessment of publication bias.

funnel plot assessment, Egger's regression

30. * Review type.

Type of review

Animal model review
No

Experimental animal exposure review
No

Pre-clinical animal intervention review
Yes

31. Language.

Select each country individually to add it to the list below, use the bin icon to remove any added in error.

English

There is not an English language summary

32. * Country.

Select the country in which the review is being carried out from the drop down list. For multi-national collaborations select all the countries involved.

United States of America

33. Other registration details.

List other places where the systematic review protocol is registered. The name of the organisation and any unique identification number assigned to the review by that organisation should be included.

n/a

34. Reference and/or URL for published protocol.

Give the citation and link for the published protocol, if there is one.

n/a

Give the link to the published protocol.

Alternatively, upload your published protocol to CRD in pdf format. Please note that by doing so you are consenting to the file being made publicly accessible.

No I do not make this file publicly available until the review is complete

Please note that the information required in the PROSPERO registration form must be completed in full even if access to a protocol is given.

35. Dissemination plans.

Give brief details of plans for communicating essential messages from the review to the appropriate audiences.

The manuscript will be submitted to a loading journal in field. In addition, a report will be submitted to the funder (Parker B Francis foundation).

Do you intend to publish the review on completion?

No

36. * Keywords.

Give words or phrases that best describe the review. Separate keywords with a semicolon or new line.

Regenerative medicine, cell-based/derived therapies, stem cells, congenital heart disease, human, clinical trials, animal studies

37. Details of any existing review of the same topic by the same authors.

Give details of earlier versions of the systematic review if an update of an existing review is being registered, including full bibliographic reference if possible.

Previous manuscript focusing on cell-based/derived therapies as a treatment for right ventricular dysfunction is currently being considered for publication.

38. * Current review status.

Review status should be updated when the review is completed and when it is published.
Please provide anticipated publication date

Review_Ongoing

39. Any additional information.

Provide any further information the review team consider relevant to the registration of the review.

40. Details of final report/publication(s).

This field should be left empty until details of the completed review are available. Give the full citation for the final report or publication of the systematic review.

Give the link to the published review.

Supplemental 2. SYRCLE criteria for animal intervention studies.

SYSTEMATIC REVIEW PROTOCOL FOR ANIMAL INTERVENTION STUDIES

FORMAT BY SYRCLE (WWW.SYRCLE.NL)
VERSION 2.0 (DECEMBER 2014)

Item #	Section/Subsection/Item	Description	Check for approval
	A. General		
1.	Title of the review	**Safety and efficacy of cell-based therapies in congenital heart disease; a systematic review and meta analysis of pre-clinical and clinical studies**	
2.	Authors (names, affiliations, contributions)	**John Martinez, MD:** conception, study design, search, data collection, protocol writing, manuscript writing<br>**Sarah Zoretic, DO:** conception, study design, search, data collection, protocol writing, manuscript writing<br>**Alvaro Moreira MD, MSc:** conception, study design, data collection and analysis, manuscript revision, supervision<br><br>University of Texas Health San Antonio<br>Department of Pediatrics, Division of Neonatology,	
3.	Other contributors (names, affiliations, contributions)	None	
4.	Contact person + e-mail address	**Alvaro Moreira**: MoreiraA@uthscsa.edu	
5.	Funding sources/sponsors	Parker B Francis Foundation	
6.	Conflicts of interest	None	
7.	Date and location of protocol registration	CAMARADES	
8.	Registration number (if applicable)	N/A	
9.	Stage of review at time of registration	Preliminary searches	
	B. Objectives		
	Background		
10.	What is already known about this disease/model/intervention? Why is it important to do this review?	Preclinical studies have established that regenerative therapies show promise as primary/adjunctive therapies for congenital heart disease (CHD). Animal models have demonstrated that regenerative cells are safe and effective. As these therapies have now translated to clinical trials in pediatric CHD, it is imperative to summarize the current findings and identify knowledge	

		gaps that still remain in order optimize translational success. Therefore, the purpose of this systematic review and meta-analysis is twofold: (i) assess the safety, and (ii) efficacy of cell-based/derived therapies in animal models of congenital heart disease.	
	Research question		
11.	Specify the disease/health problem of interest	Congenital heart disease: Hypoplastic left heart syndrome, Tricuspid atresia, Single ventricle physiology, Transposition of great arteries, Tetralogy of Fallot, Pulmonary Atresia, Anomalous pulmonary venous return, Double outlet right ventricle, Single inlet ventricle, Coarctation of aorta, Interrupted aortic arch, Ebstein's anomaly.	
12.	Specify the population/species studied	Animal models of congenital heart disease (as listed above)	
13.	Specify the intervention/exposure	Cell-based/derived therapies:<br>mesenchymal, embryonic, multipotent, inducible pluripotent cells, progenitor, hematopoietic, umbilical cord, cord blood, c-kit+, secretome, exosome, microRNA, microvesicle, extracellular vesicle.	
14.	Specify the control population	Placebo or no treatment	
15.	Specify the outcome measures	Primary Outcome: Safety and cardiac function (refer to number 26)	
16.	State your research question (based on items 11-15)	Are cell-based/derived therapies both safe and efficacious in experimental models of congenital heart disease?	
	C. Methods		
	Search and study identification		
17.	Identify literature databases to search (*e.g.* Pubmed, Embase, Web of science)	X MEDLINE via PubMed X Web of Science<br>X SCOPUS ☐ EMBASE<br>X Other, namely: Science direct<br>☐ Specific journal(s), namely:	
18.	Define electronic search strategies (*e.g.* use the step by step search guide[15] and animal search filters[20, 21])	When available, please add a supplementary file containing your search strategy: [insert file name]	
19.	Identify other sources for study identification	X Reference lists of included studies ☐ Books<br>X Reference lists of relevant reviews<br>☐ Conference proceedings, namely:<br>☐ Contacting authors/ organisations, namely:<br>☐ Other, namely:	
20.	Define search strategy for these other sources	Screening the reference lists for relevant titles and screening the abstracts of these relevant titles	
	Study selection		

21.	Define screening phases (*e.g.* pre-screening based on title/abstract, full text screening, both)	First phase: screening by title and abstract<br>Second phase: full text screening of eligible articles<br>Full text studies that do not meet inclusion will be incorporated into the flow diagram with reasons for exclusion	
22.	Specify (a) the number of reviewers per screening phase and (b) how discrepancies will be resolved	a) Two investigators (J. Martinez & S. Zoretic) will independently screen all the abstracts/full texts for the inclusion criteria. b) Differences of opinion in either phase that cannot be resolved by discussion will be resolved by consulting a third investigator (A. Moreira).	
	Define all inclusion and exclusion criteria based on:		
23.	Type of study (design)	Inclusion criteria: pre-clinical studies<br>Exclusion criteria: non-intervention studies, no control group, co-intervention studies	
24.	Type of animals/population (*e.g.* age, gender, disease model)	Inclusion criteria: animal models of congenital heart disease, all genders<br>Exclusion criteria: humans, in-vitro, non-pediatric models of heart disease	
25.	Type of intervention (*e.g.* dosage, timing, frequency)	Inclusion criteria: administration of cell-based/derived therapy- all dosages, timing, and frequency; cells may be derived from any tissue source<br>Exclusion criteria: Cardiac administration of cell-based/derived therapy assessing for variables other than safety or effect on function.	
26.	Outcome measures	<u>Cardiac function as measured by:</u><br>-Right/Left ejection fraction<br>-End diastolic volume<br>-End systolic volume<br>-Tricuspid annular plane systolic excursion<br>-Fractional area change<br>-Fractional shortening<br><br><u>Safety:</u><br>-Mortality<br>-Adverse events with administration (fever, rash, infection, hemodynamic instability, arrhythmias, etc)<br><br>Exclusion criteria:	
27.	Language restrictions	Inclusion criteria: English and Spanish<br>Exclusion criteria: All other languages	
28.	Publication date restrictions	Inclusion criteria: no publication date restrictions<br>Exclusion criteria:	
29.	Other	Inclusion criteria:<br>Exclusion criteria:	
30.	Sort and prioritize your exclusion criteria per selection phase	<u>Selection phase:</u> title and abstract screening<br>1. Not a primary study<br>2. Not an in vivo animal study	

		3. Not congenital heart disease<br>4. No cell based/derived therapy use<br>5. Adult animal<br><br><u>Selection phase:</u> full text screening<br>1. Not a primary study<br>2. Not an in vivo animal study<br>3. Not congenital heart disease<br>4. No cell based/derived therapy use<br>5. No assessment of safety, effect on ventricular function<br>5. No control group<br>6. Co-intervention studies	
	Study characteristics to be extracted (for assessment of external validity, reporting quality)		
31.	Study ID (*e.g.* authors, year)	Authors, journal, title, year, language, contact author e-mail	
32.	Study design characteristics (*e.g.* experimental groups, number of animals)	Number of animals in experimental and control groups, reporting of randomization process, power calculation reported, method(s) to induce congenital heart disease	
33.	Animal model characteristics (*e.g.* species, gender, disease induction)	Animal species, strain, age, gender, weight, and immune status	
34.	Intervention characteristics (*e.g.* intervention, timing, duration)	Source, dose, delivery, timing, and frequency of intervention	
35.	Outcome measures	Assessment of safety as defined through mortality or occurrence of adverse events upon administration.	
36.	Other (*e.g.* drop-outs)	Assessment of cardiac function through measures as noted above	
	Assessment risk of bias (internal validity) or study quality		
37.	Specify (a) the number of reviewers assessing the risk of bias/study quality in each study and (b) how discrepancies will be resolved	a) Two investigators (J. Martinez & S. Zoretic) will independently screen all the abstracts/full texts for the inclusion criteria. b) Differences of opinion in either phase that cannot be resolved by discussion will be resolved by consulting a third investigator (A. Moreira)	
38.	Define criteria to assess (a) the internal validity of included studies (*e.g.* selection, performance, detection and attrition bias) and/or (b) other study quality measures (*e.g.* reporting quality, power)	X By use of SYRCLE's Risk of Bias tool[4]<br>☐By use of SYRCLE's Risk of Bias tool, adapted as follows:<br>☐By use of CAMARADES' study quality checklist, e.g [22]<br>☐By use of CAMARADES' study quality checklist, adapted as follows:<br>☐Other criteria, namely:	
	Collection of outcome data		
39.	For each outcome measure, define the type of data to be extracted (*e.g.* continuous/dichotomous, unit of measurement)	All outcome measures will be expressed through study units of measure, values expressed as continuous measures will be recorded as means +/- SD, SEM or median +/- IQR	

40.	Methods for data extraction/retrieval (*e.g.* first extraction from graphs using a digital screen ruler, then contacting authors)	Extraction from text, tables, and figures (GetData graph digitizer 2.26)<br>Contact authors in case of missing data	
41.	Specify (a) the number of reviewers extracting data and (b) how discrepancies will be resolved	a) Two investigators (J. Martinez & S. Zoretic) will independently screen all the abstracts/full texts for the inclusion criteria. b) Differences of opinion in either phase that cannot be resolved by discussion will be resolved by consulting a third investigator (A. Moreira)	
	Data analysis/synthesis		
42.	Specify (per outcome measure) how you are planning to combine/compare the data (*e.g.* descriptive summary, meta-analysis)	For sufficient data, we will conduct a meta-analysis for eligible studies. If insufficient data to measure outcomes, we will provide a descriptive summary of study results	
43.	Specify (per outcome measure) how it will be decided whether a meta-analysis will be performed	A minimum of 4 articles for the same outcome is required. High heterogeneity is expected between studies due to differences in the study designs. We will perform a meta-regression analysis to investigate sources of heterogeneity.	
	If a meta-analysis seems feasible/sensible, specify (for each outcome measure):		
44.	The effect measure to be used (*e.g.* mean difference, standardized mean difference, risk ratio, odds ratio)	Continuous outcomes will be analysed using standardized mean differences (95% CI)	
45.	The statistical model of analysis (*e.g.* random or fixed effects model)	Random effects model	
46.	The statistical methods to assess heterogeneity (*e.g.* I^2, Q)	I^2	
47.	Which study characteristics will be examined as potential source of heterogeneity (subgroup analysis)	Study design: experimental and control groups, congenital heart disease model, measures of safety, measures of cardiac function.<br>Animal model: species, strain, age, gender<br>Cell based/derived therapy source: dose, delivery, timing, frequency, transplant method (allogeneic, xenogeneic, autologous, etc.)	
48.	Any sensitivity analyses you propose to perform	If high heterogeneity is observed (≥70%), subgroup analyses will be conducted	
49.	Other details meta-analysis (*e.g.* correction for multiple testing, correction for multiple use of control group)	N/A	
50.	The method for assessment of publication bias	Funnel plot assessment<br>Egger's regression	
Final approval by (names, affiliations):		John Martinez MD<br>Sarah Zoretic DO<br>Alvaro Moreira MD, MSc	Date:

UT Health San Antonio
Department of Pediatrics, Division of Neonatology
7703 Floyd Curl Drive MC 7812
San Antonio, TX, USA 78229

Supplemental 3. Database search terms.

Database Search Terms:

("regenerative" OR "stem cell" OR "stromal cell" OR "mesenchymal" OR "embryonic" OR "pluripotent" OR "multipotent" OR "inducible pluripotent" OR "progenitor" OR "hematopoietic" OR "umbilical cord" OR "cord blood" OR "microparticle" OR "extracellular vessicles") AND (“tetralogy of fallot” or “single ventricle” or “transposition of great arteries” or “anomalous pulmonary venous” or “tricuspid atresia” or “truncus arteriosus” or “hypoplastic left heart” or “ebstein” or “double outlet right ventricle” or “hypoplastic right heart” or “pulmonary atresia” or “coarctation of aorta” or “interrupted aortic arch” or “single inlet ventricle”)

Supplemental 4. List of included studies.

1. Agarwal U, Smith AW, French KM, Boopathy A V., George A, Trac D, Brown ME, Shen M, Jiang R, Fernandez JD, Kogon BE, Kanter KR, Alsoufi B, Wagner MB, Platt MO, Davis ME. Age-Dependent Effect of Pediatric Cardiac Progenitor Cells After Juvenile Heart Failure. *Stem Cells Transl Med* 2016;**5**:883–892.
2. Albertario A, Swim MM, Ahmed EM, Iacobazzi D, Yeong M, Madeddu P, Ghorbel MT, Caputo M. Successful Reconstruction of the Right Ventricular Outflow Tract by Implantation of Thymus Stem Cell Engineered Graft in Growing Swine. *JACC Basic to Transl Sci* 2019;**4**:364–384.
3. Borenstein N, Jian Z, Fromont G, Bruneval P, Hekmati M, Behr L, Laborde F, Montarras D, Bret E Le. Noncultured cell transplantation in an ovine model of right ventricular preparation. *J Thorac Cardiovasc Surg* 2005;**129**:1119–1127.
4. Brizard CP, Looi JYJ, Smolich JJ, Horton SB, Angerosa J, Elwood NJ, Pepe S. Safety of intracoronary human cord blood stem cells in a lamb model of infant cardiopulmonary bypass. *Ann Thorac Surg* The Society of Thoracic Surgeons; 2015;**100**:1021–1029.
5. Burkhart HM, Qureshi MY, Peral SC, O'Leary PW, Olson TM, Cetta F, Nelson TJ. Regenerative therapy for hypoplastic left heart syndrome: First report of intraoperative intramyocardial injection of autologous umbilical-cord blood-derived cells. *J Thorac Cardiovasc Surg* Elsevier Inc.; 2015;**149**:e35–e37.
6. Burkhart HM, Qureshi MY, Rossano JW, Cantero Peral S, O'Leary PW, Hathcock M, Kremers W, Nelson TJ, Breuer A, Cavanaugh K, Cetta F, Dearani JA, Dietz A, Edwards B, Hirsch S, Holst K, Krucker K, Lenn K, Martineau S, Mascio CE, Majerus A, Miller A, Miller J, Miller K, Mir A, Olson TM, Radel D, Reece C, Riess L, Said SM, et al. Autologous stem cell therapy for hypoplastic left heart syndrome: Safety and feasibility of intraoperative intramyocardial injections. *J Thorac Cardiovasc Surg* 2019;
7. Cao G, Liu C, Wan Z, Liu K, Sun H, Sun X, Tang M, Bing W, Wu S, Pang X, Zhang X. Combined hypoxia inducible factor-1α and homogeneous endothelial progenitor cell therapy attenuates shunt flow-induced pulmonary arterial hypertension in rabbits. *J Thorac Cardiovasc Surg* Elsevier Inc.; 2015;**150**:621–632.
8. Chery J, Huang S, Gong L, Wang S, Yuan Z, Wong J, Lee J, Johnson S, Si MS. Human neonatal thymus mesenchymal stem/stromal cells and chronic right ventricle pressure overload. *Bioengineering* 2019;**6**:1–9.
9. Davies B, Elwood NJ, Li S, Cullinane F, Edwards GA, Newgreen DF, Brizard CP. Human Cord Blood Stem Cells Enhance Neonatal Right Ventricular Function in an Ovine Model of Right Ventricular Training. *Ann Thorac Surg* Elsevier Inc.; 2010;**89**:585-593.e4.
10. Eitoku T, Baba K, Kondou M, Kurita Y, Fukushima Y, Hirai K, Ohtsuki S, Ishigami S, Sano S, Oh H. Transcoronary cell infusion with the stop-flow technique in children with single-ventricle physiology. *Pediatr Int* 2018;**60**:240–246.
11. Henning RJ, Aufman J, Shariff M, Sawmiller D, Delostia V, Sanberg P, Morgan M. Human umbilical cord blood mononuclear cells decrease fibrosis and increase cardiac function in cardiomyopathy. *Regen Med* 2010;**5**:45–54.
12. Ishigami S, Ohtsuki S, Tarui S, Ousaka D, Eitoku T, Kondo M, Okuyama M, Kobayashi J, Baba K, Arai S, Kawabata T, Yoshizumi K, Tateishi A, Kuroko Y, Iwasaki T, Sato S, Kasahara S, Sano S, Oh H. Intracoronary autologous cardiac progenitor cell transfer in patients with hypoplastic left heart syndrome: The TICAP Prospective Phase 1 Controlled

Trial. *Circ Res* 2015;**116**:653–664.

13. Ishigami S, Ohtsuki S, Eitoku T, Ousaka D, Kondo M, Kurita Y, Hirai K, Fukushima Y, Baba K, Goto T, Horio N, Kobayashi J, Kuroko Y, Kotani Y, Arai S, Iwasaki T, Sato S, Kasahara S, Sano S, Oh H. Intracoronary cardiac progenitor cells in single ventricle physiology: the perseus (cardiac progenitor cell infusion to treat univentricular heart disease) randomized phase 2 trial. *Circ Res* 2017;**120**:1162–1173.
14. Pincott ES, Ridout D, Brocklesby M, McEwan A, Muthurangu V, Burch M. A randomized study of autologous bone marrow–derived stem cells in pediatric cardiomyopathy. *J Hear Lung Transplant* Elsevier Inc.; 2017;**36**:837–844.
15. Lambert V, Gouadon E, Capderou A, Bret E Le, Ly M, Dinanian S, Renaud JF, Pucéat M, Rücker-Martin C. Right ventricular failure secondary to chronic overload in congenital heart diseases: Benefits of cell therapy using human embryonic stem cell-derived cardiac progenitors. *J Thorac Cardiovasc Surg* 2015;**149**:708-715.e1.
16. Liu K, Liu R, Cao G, Sun H, Wang X, Wu S. Adipose-Derived Stromal Cell Autologous Transplantation. 2011;**20**.
17. Nana-Leventaki E, Nana M, Poulianitis N, Sampaziotis D, Perrea D, Sanoudou D, Rontogianni D, Malliaras K. Cardiosphere-Derived Cells Attenuate Inflammation, Preserve Systolic Function, and Prevent Adverse Remodeling in Rat Hearts With Experimental Autoimmune Myocarditis. *J Cardiovasc Pharmacol Ther* 2019;**24**:70–77.
18. Qureshi MY, Cabalka AK, Khan SP, Hagler DJ, Haile DT, Cannon BC, Olson TM, Cantero-Peral S, Dietz AB, Radel DJ, Taggart NW, Kelle AM, Rodriguez V, Dearani JA, O'Leary PW, Nelson TJ, Cavanaugh KM, Miller JM, Miller KS. Cell-Based Therapy for Myocardial Dysfunction After Fontan Operation in Hypoplastic Left Heart Syndrome. *Mayo Clin Proc Innov Qual Outcomes* 2017;**1**:185–191.
19. Rivas J, Menéndez JJ, Arrieta R, Alves J, Romero MP, García-Guereta L, Álvarez-Doforno R, Parrón M, González A, Ruza F, Gutiérrez-Larraya F. Utilidad de la terapia intracoronaria con células progenitoras en pacientes con miocardiopatía dilatada: ¿Puente o alternativa al trasplante cardiaco? *An Pediatr* 2011;**74**:218–225.
20. Rupp S, Zeiher AM, Dimmeler S, Tonn T, Bauer J, Jux C, Akintuerk H, Schranz D. A regenerative strategy for heart failure in hypoplastic left heart syndrome: Intracoronary administration of autologous bone marrow-derived progenitor cells. *J Hear Lung Transplant* Elsevier Inc.; 2010;**29**:574–577.
21. Rupp S, Jux C, Bönig H, Bauer J, Tonn T, Seifried E, Dimmeler S, Zeiher AM, Schranz D. Intracoronary bone marrow cell application for terminal heart failure in children. *Cardiol Young* 2012;**22**:558–563.
22. Sano T, Ousaka D, Goto T, Ishigami S, Hirai K, Kasahara S, Ohtsuki S, Sano S, Oh H. Impact of Cardiac Progenitor Cells on Heart Failure and Survival in Single Ventricle Congenital Heart Disease. *Circ Res* 2018;**122**:994–1005.
23. Schmuck EG, Hacker TA, Schreier DA, Chesler NC, Wang Z. Beneficial effects of mesenchymal stem cell delivery via a novel cardiac bioscaffold on right ventricles of pulmonary arterial hypertensive rats. *Am J Physiol - Hear Circ Physiol* 2019;**316**:H1005–H1013.
24. Sugiura T, Hibino N, Breuer CK, Shinoka T. Tissue-engineered cardiac patch seeded with human induced pluripotent stem cell derived cardiomyocytes promoted the regeneration of host cardiomyocytes in a rat model. *J Cardiothorac Surg* Journal of Cardiothoracic Surgery; 2016;**11**:1–8.

25. Tarui S, Ishigami S, Ousaka D, Kasahara S, Ohtsuki S, Sano S, Oh H. Transcoronary infusion of cardiac progenitor cells in hypoplastic left heart syndrome: Three-year follow-up of the Transcoronary Infusion of Cardiac Progenitor Cells in Patients with Single-Ventricle Physiology (TICAP) trial. *J Thorac Cardiovasc Surg* Elsevier; 2015;**150**:1198-1208.e2.
26. Trac D, Maxwell JT, Brown ME, Xu C, Davis ME. Aggregation of Child Cardiac Progenitor Cells Into Spheres Activates Notch Signaling and Improves Treatment of Right Ventricular Heart Failure. *Circ Res* 2019;**124**:526–538.
27. Umar S, Visser YP De, Steendijk P, Schutte CI, Laghmani EH, Wagenaar GTM, Bax WH, Mantikou E, Pijnappels DA, Atsma DE, Schalij MJ, Wall EE Van Der, Laarse A Van Der. Allogenic stem cell therapy improves right ventricular function by improving lung pathology in rats with pulmonary hypertension. *Am J Physiol - Hear Circ Physiol* 2009;**297**:1606–1616.
28. Wehman B, Sharma S, Pietris N, Mishra R, Siddiqui OT, Bigham G, Li T, Aiello E, Murthi S, Pittenger M, Griffith B, Kaushal S. Mesenchymal stem cells preserve neonatal right ventricular function in a porcine model of pressure overload. *Am J Physiol - Hear Circ Physiol* 2016;**310**:H1816–H1826.
29. Wehman B, Pietris N, Bigham G, Siddiqui O, Mishra R, Li T, Aiello E, Jack G, Wang W, Murthi S, Sharma S, Kaushal S. Cardiac Progenitor Cells Enhance Neonatal Right Ventricular Function After Pulmonary Artery Banding. *Ann Thorac Surg* The Society of Thoracic Surgeons; 2017;**104**:2045–2053.
30. Yerebakan C, Sandica E, Prietz S, Klopsch C, Ugurlucan M, Kaminski A, Abdija S, Lorenzen B, Boltze J, Nitzsche B, Egger D, Barten M, Furlani D, Ma N, Vollmar B, Liebold A, Steinhoff G. Autologous umbilical cord blood mononuclear cell transplantation preserves right ventricular function in a novel model of chronic right ventricular volume overload. *Cell Transplant* 2009;**18**:855–868.
31. Zschirnt M, Jux C, Boenig H, Zeiher A, Assmus B, Khalil M, Kriebel T, Rupp S. Neonatal myocardial infarction: substantial improvement of cardiac function after autologous bone marrow-derived cell therapy. *Clin Res Cardiol* Springer Berlin Heidelberg; 2019;2–4.

Supplemental 5. PRISMA Checklist.

PRISMA 2009 Checklist

Section/topic	#	Checklist item	Reported on page #
**TITLE**			
Title	1	Identify the report as a systematic review, meta-analysis, or both.	2
**ABSTRACT**			
Structured summary	2	Provide a structured summary including, as applicable: background; objectives; data sources; study eligibility criteria, participants, and interventions; study appraisal and synthesis methods; results; limitations; conclusions and implications of key findings; systematic review registration number.	3
**INTRODUCTION**			
Rationale	3	Describe the rationale for the review in the context of what is already known.	4
Objectives	4	Provide an explicit statement of questions being addressed with reference to participants, interventions, comparisons, outcomes, and study design (PICOS).	4
**METHODS**			
Protocol and registration	5	Indicate if a review protocol exists, if and where it can be accessed (e.g., Web address), and, if available, provide registration information including registration number.	5
Eligibility criteria	6	Specify study characteristics (e.g., PICOS, length of follow-up) and report characteristics (e.g., years considered, language, publication status) used as criteria for eligibility, giving rationale.	5-6
Information sources	7	Describe all information sources (e.g., databases with dates of coverage, contact with study authors to identify additional studies) in the search and date last searched.	5
Search	8	Present full electronic search strategy for at least one database, including any limits used, such that it could be repeated.	5
Study selection	9	State the process for selecting studies (i.e., screening, eligibility, included in systematic review, and, if applicable, included in the meta-analysis).	5
Data collection process	10	Describe method of data extraction from reports (e.g., piloted forms, independently, in duplicate) and any processes for obtaining and confirming data from investigators.	5-6
Data items	11	List and define all variables for which data were sought (e.g., PICOS, funding sources) and any assumptions and simplifications made.	5-6
Risk of bias in individual studies	12	Describe methods used for assessing risk of bias of individual studies (including specification of whether this was done at the study or outcome level), and how this information is to be used in any data synthesis.	8
Summary measures	13	State the principal summary measures (e.g., risk ratio, difference in means).	7-8
Synthesis of results	14	Describe the methods of handling data and combining results of studies, if done, including measures of consistency (e.g., I^2) for each meta-analysis.	7-8

PRISMA 2009 Checklist

Section/topic	#	Checklist item	Reported on page #
Risk of bias across studies	15	Specify any assessment of risk of bias that may affect the cumulative evidence (e.g., publication bias, selective reporting within studies).	8
Additional analyses	16	Describe methods of additional analyses (e.g., sensitivity or subgroup analyses, meta-regression), if done, indicating which were pre-specified.	7-8
**RESULTS**			
Study selection	17	Give numbers of studies screened, assessed for eligibility, and included in the review, with reasons for exclusions at each stage, ideally with a flow diagram.	9
Study characteristics	18	For each study, present characteristics for which data were extracted (e.g., study size, PICOS, follow-up period) and provide the citations.	9, 11
Risk of bias within studies	19	Present data on risk of bias of each study and, if available, any outcome level assessment (see item 12).	13-14
Results of individual studies	20	For all outcomes considered (benefits or harms), present, for each study: (a) simple summary data for each intervention group (b) effect estimates and confidence intervals, ideally with a forest plot.	9-13
Synthesis of results	21	Present results of each meta-analysis done, including confidence intervals and measures of consistency.	9-13
Risk of bias across studies	22	Present results of any assessment of risk of bias across studies (see Item 15).	13-14
Additional analysis	23	Give results of additional analyses, if done (e.g., sensitivity or subgroup analyses, meta-regression [see Item 16]).	10-11, 12-13
**DISCUSSION**			
Summary of evidence	24	Summarize the main findings including the strength of evidence for each main outcome; consider their relevance to key groups (e.g., healthcare providers, users, and policy makers).	15-18
Limitations	25	Discuss limitations at study and outcome level (e.g., risk of bias), and at review-level (e.g., incomplete retrieval of identified research, reporting bias).	18
Conclusions	26	Provide a general interpretation of the results in the context of other evidence, and implications for future research.	19
**FUNDING**			
Funding	27	Describe sources of funding for the systematic review and other support (e.g., supply of data); role of funders for the systematic review.	19

From: Moher D, Liberati A, Tetzlaff J, Altman DG, The PRISMA Group (2009). Preferred Reporting Items for Systematic Reviews and Meta-Analyses: The PRISMA Statement. PLoS Med 6(7): e1000097. doi:10.1371/journal.pmed1000097

For more information, visit: **www.prisma-statement.org**.

Page 2 of 2

Supplemental 6. **Effect size of regenerative cell on animal ejection fraction.** Forest plots demonstrating MD and 95% CI for A) Left ventricular ejection fraction; cell-based n=172; control=177; p <0.0001. B) Right ventricular ejection fraction; cell-based n=84; control n=84; p=0.02. C) Disease model; cell-based n= 256; control n=261; RVHF, p=0.01; DCM, p<0.0001.

	Experimental			Control					
**Study**	**Total**	**Mean**	**SD**	**Total**	**Mean**	**SD**	**MD**	**95%–CI**	**Weight**
Henning 2010	22	53.50	8.00	23	41.70	14.80	11.80	[4.89; 18.71]	9.7%
Henning 2010	22	49.80	16.90	23	44.90	9.10	4.90	[–3.08; 12.88]	7.7%
Henning 2010	12	57.00	9.40	10	41.60	9.80	15.40	[7.33; 23.47]	7.6%
Henning 2010	12	52.30	11.80	10	43.10	5.40	9.20	[1.73; 16.67]	8.6%
Henning 2010	22	87.50	6.10	23	83.80	8.60	3.70	[–0.64; 8.04]	18.4%
Henning 2010	22	76.00	11.30	23	66.70	6.70	9.30	[3.84; 14.76]	13.7%
Henning 2010	22	64.20	6.10	23	57.20	6.20	7.00	[3.41; 10.59]	22.4%
Nana 2019	6	81.50	7.40	8	65.40	22.60	16.10	[–0.64; 32.84]	2.0%
Nana 2019	6	78.30	7.40	8	78.40	11.30	–0.10	[–9.92; 9.72]	5.4%
Suguira 2016	10	74.30	21.40	10	73.60	11.30	0.70	[–14.30; 15.70]	2.5%
Suguira 2016	10	72.90	28.20	10	73.60	18.10	–0.70	[–21.47; 20.07]	1.3%
Yerebakan 2009	6	72.70	7.80	6	60.30	43.40	12.40	[–22.88; 47.68]	0.5%
**Random effects model**	**172**			**177**			**7.41**	**[4.96; 9.85]**	**100.0%**

Heterogeneity: $I^2 = 21\%$, $\tau^2 = 3.5835$, $p = 0.24$

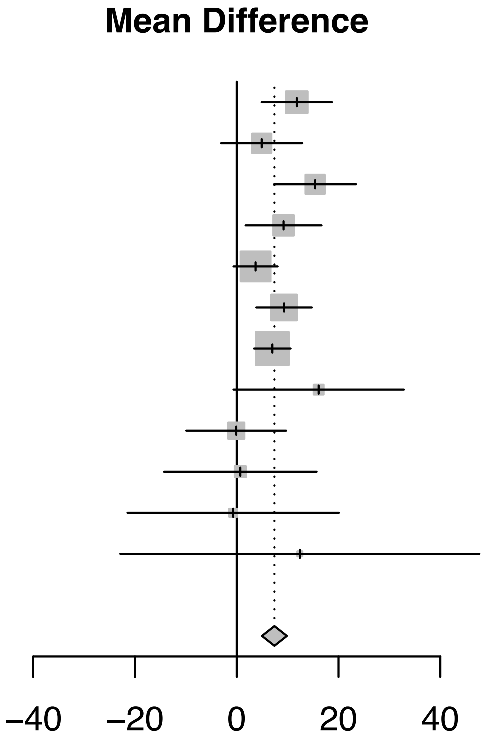

Supplemental 6. A) Cell-based effect on animal LVEF.

Study	Experimental Total	Experimental Mean	Experimental SD	Control Total	Control Mean	Control SD	MD	95%–CI	Weight
Agarwal 2016	6	64.60	5.90	5	51.00	6.30	13.60	[6.33; 20.87]	10.7%
Agarwal 2016	6	62.80	5.40	5	48.30	4.50	14.50	[8.65; 20.35]	11.6%
Albertario 2019	5	76.00	8.90	5	83.00	13.40	−7.00	[−21.10; 7.10]	6.5%
Davies 2010	8	49.70	3.90	8	52.30	3.90	−2.60	[−6.42; 1.22]	12.8%
Lambert 2015	6	48.00	4.80	6	41.60	8.90	6.40	[−1.69; 14.49]	10.1%
Lambert 2015	6	49.50	13.20	6	43.30	13.90	6.20	[−9.14; 21.54]	5.9%
Yerebakan 2009	9	54.60	12.60	9	58.50	13.20	−3.90	[−15.82; 8.02]	7.7%
Yerebakan 2009	7	66.90	13.50	8	61.60	13.60	5.30	[−8.44; 19.04]	6.7%
Yerebakan 2009	7	74.80	11.60	8	69.50	13.90	5.30	[−7.61; 18.21]	7.1%
Yerebakan 2009	6	73.90	22.80	6	62.60	29.40	11.30	[−18.47; 41.07]	2.3%
Umar	10	52.10	5.20	10	42.80	6.20	9.30	[4.28; 14.32]	12.1%
Schmuck	8	63.00	12.00	8	49.00	16.00	14.00	[0.14; 27.86]	6.6%
**Random effects model**	**84**			**84**			**6.10**	**[1.17; 11.03]**	**100.0%**

Heterogeneity: $I^2 = 73\%$, $\tau^2 = 45.6731$, $p < 0.01$

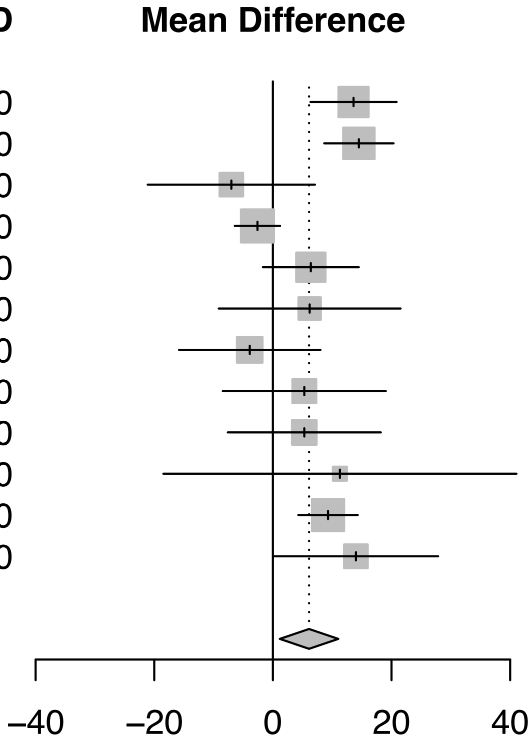

Supplemental 6. B) Cell-based effect on animal RVEF.

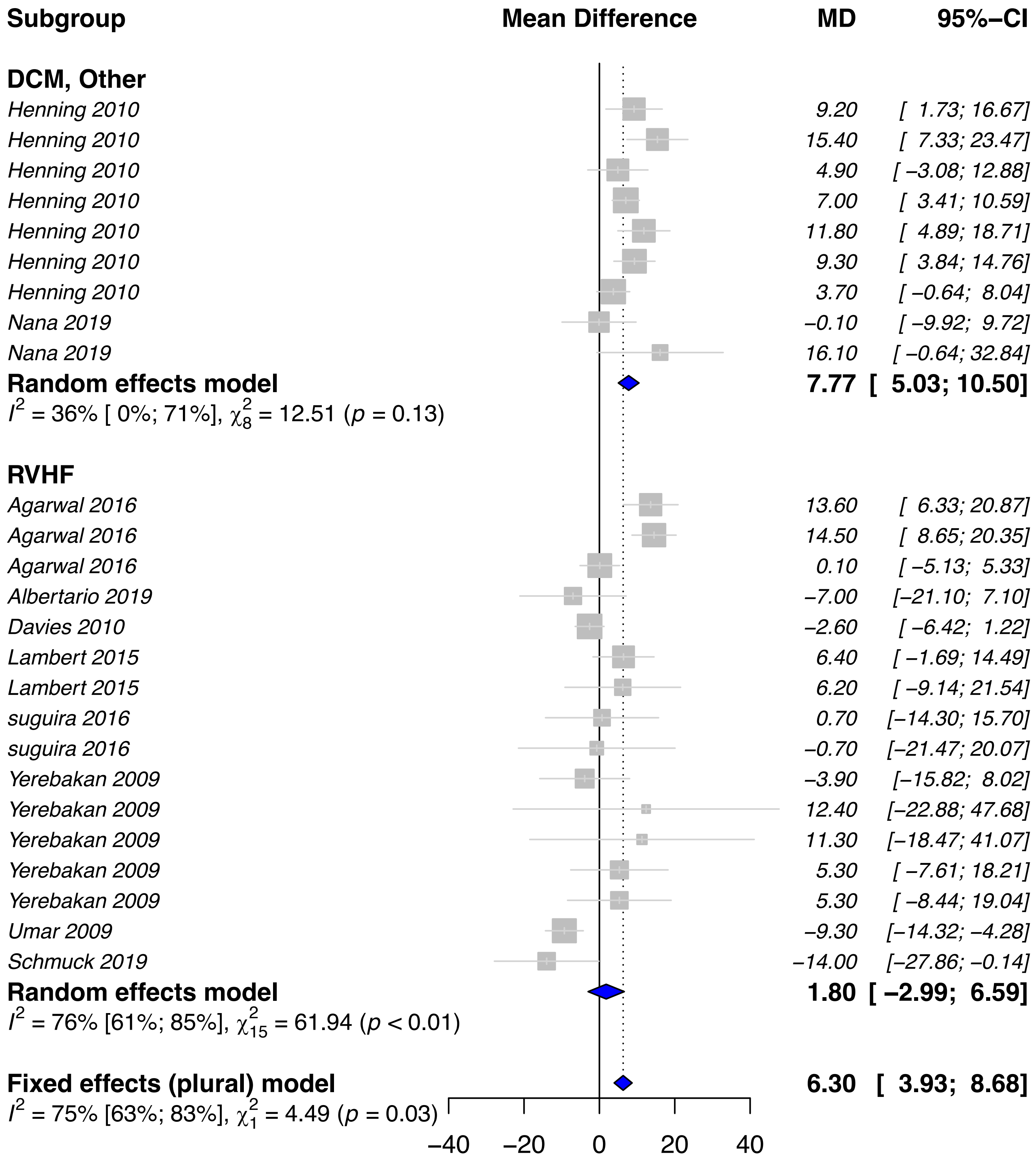

Supplemental 6. C) Cell-based effect on animal ejection fraction by disease model.

Supplemental 7. Subgroup analysis of regenerative cell effect size on animal ejection fraction. Forest plots demonstrating MD and 95% CI for A) Route of delivery, p<0.00001 for intramyocardial injection. B) Dose, p<0.00001 for 1-10 M. C) Tissue Source, p<0.0001 for cardiac; p=0.0003 for bone marrow. D) Timing of delivery, p<0.0001 for 1 week–1 month. E) autologous vs. non-autologous sources, p<0.0001 (non-autologous). Cell-based n=256; Control n=261.

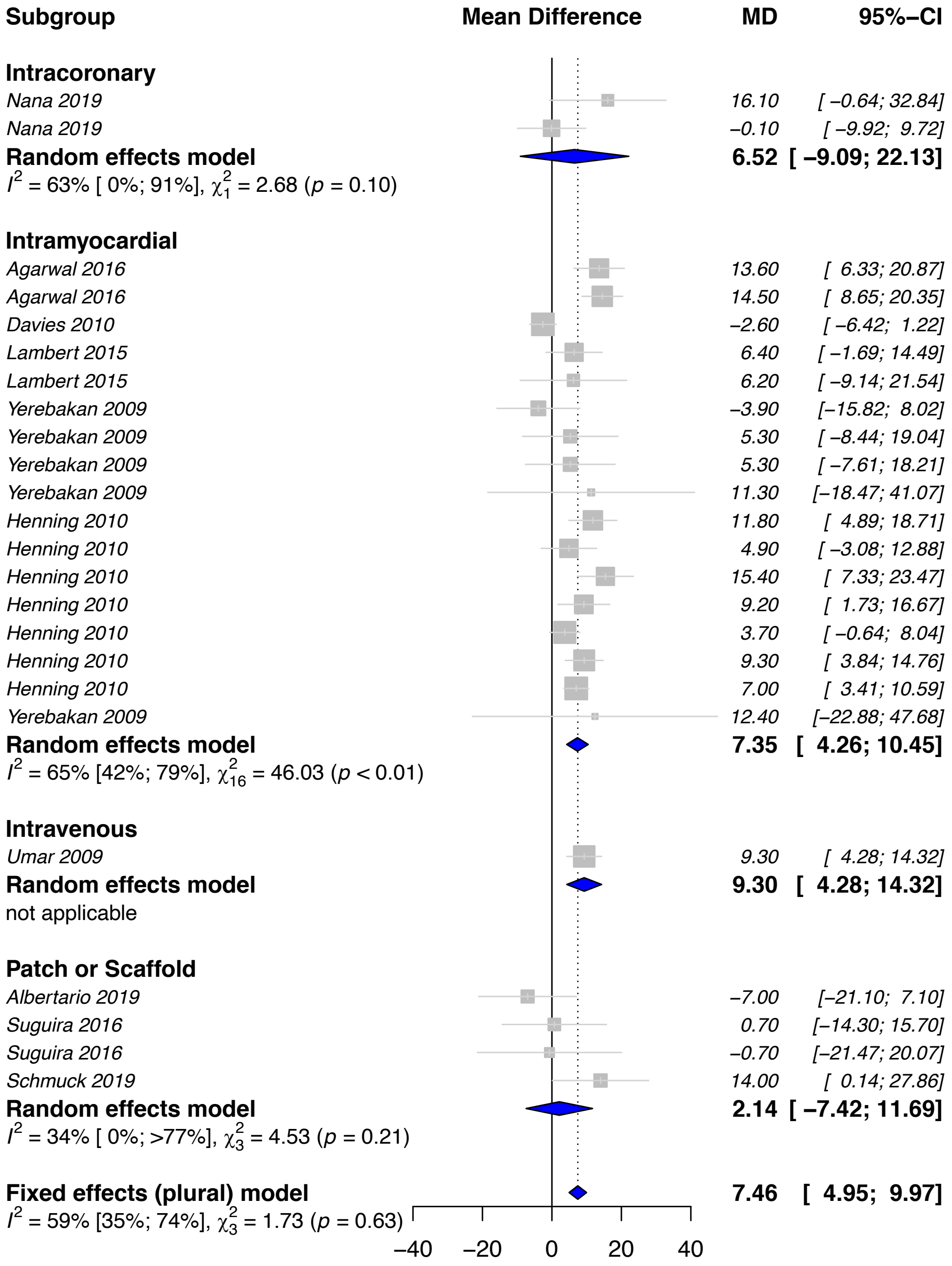

Supplemental 7. A) Cell-based effect on animal ejection fraction by route of delivery.

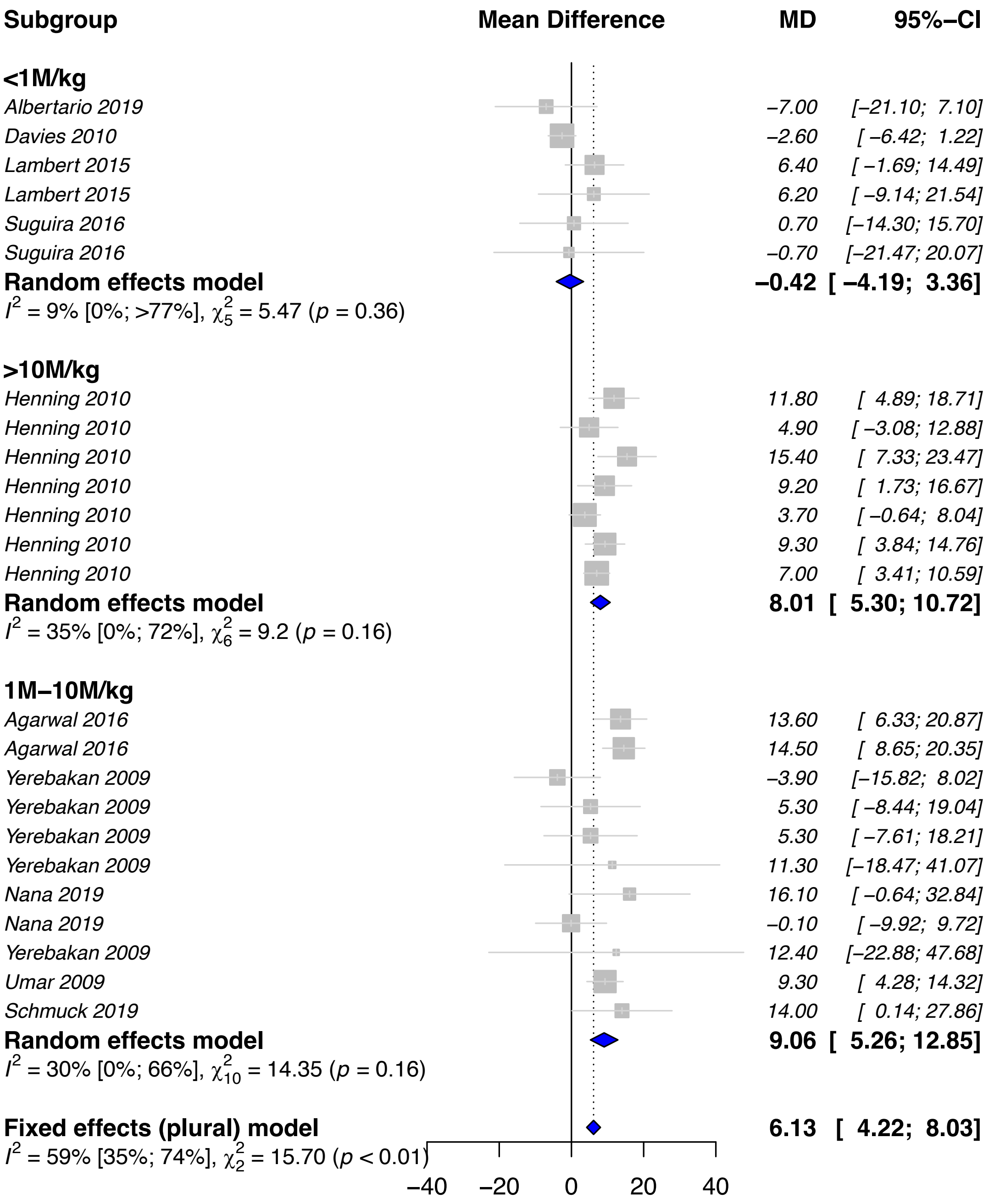

Supplemental 7. B) Cell-based effect on animal ejection fraction by dose.

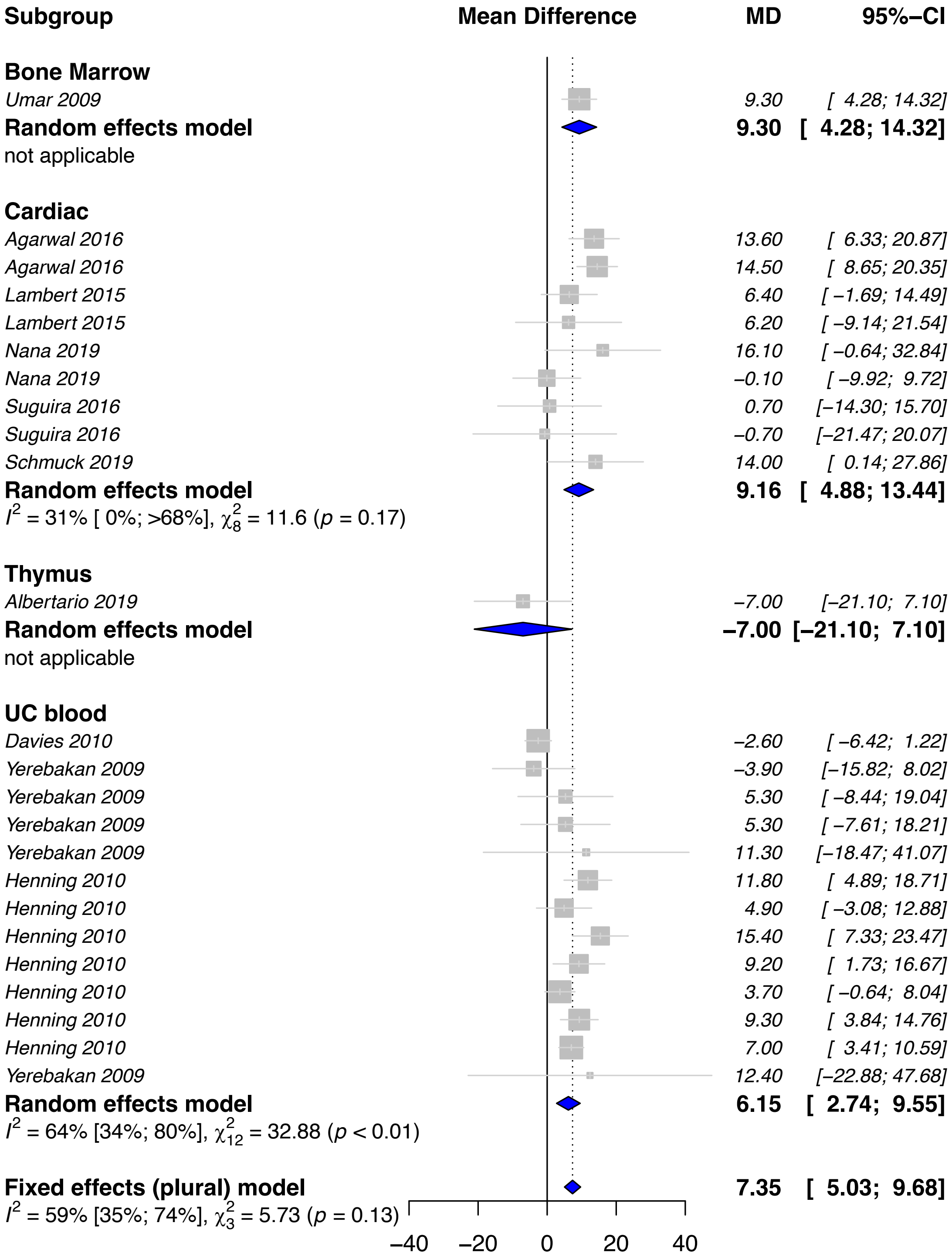

Supplemental 7. C) Cell-based effect on animal ejection fraction by tissue source.

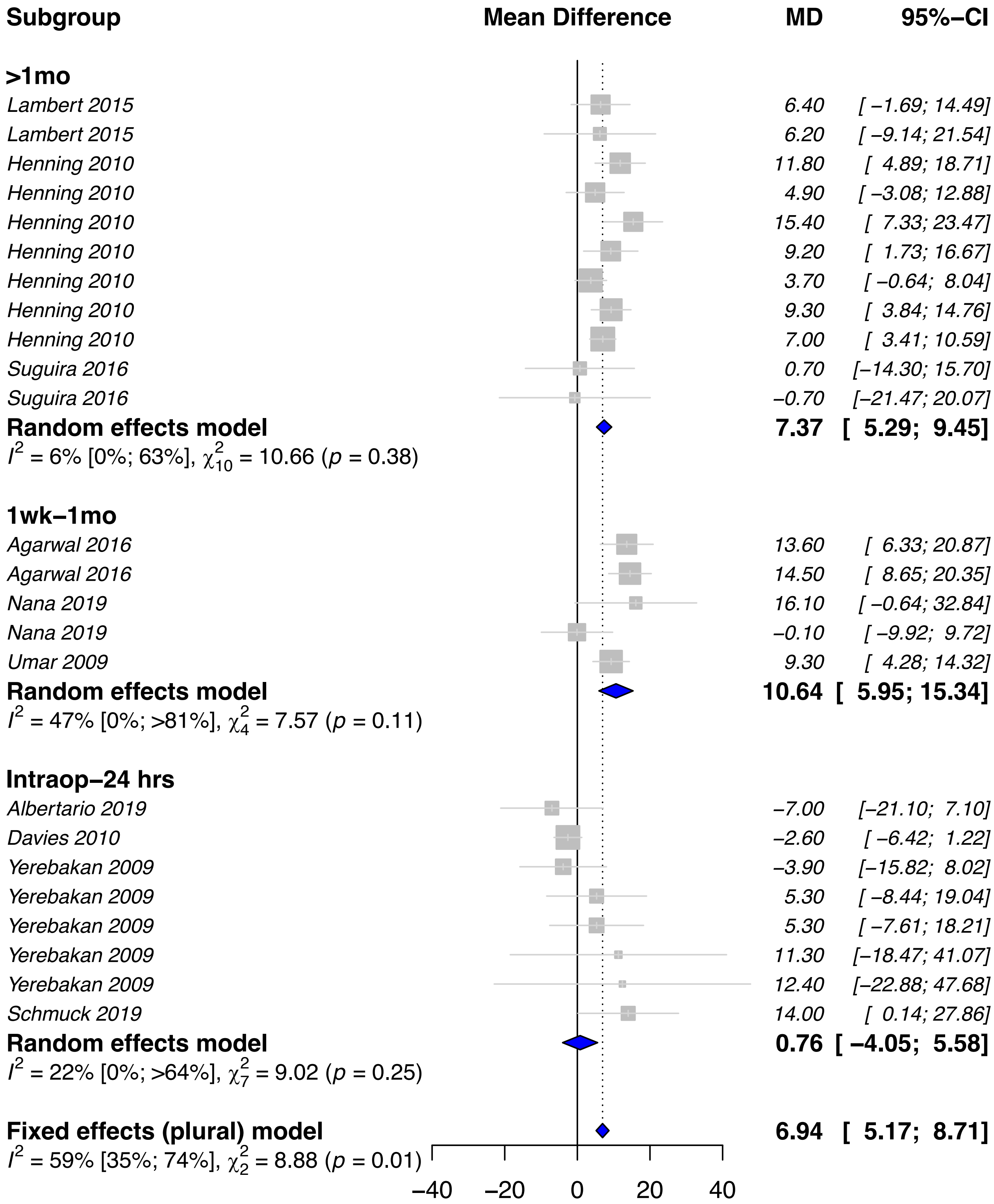

Supplemental 7. D) Cell-based effect on animal ejection fraction by timing of delivery.

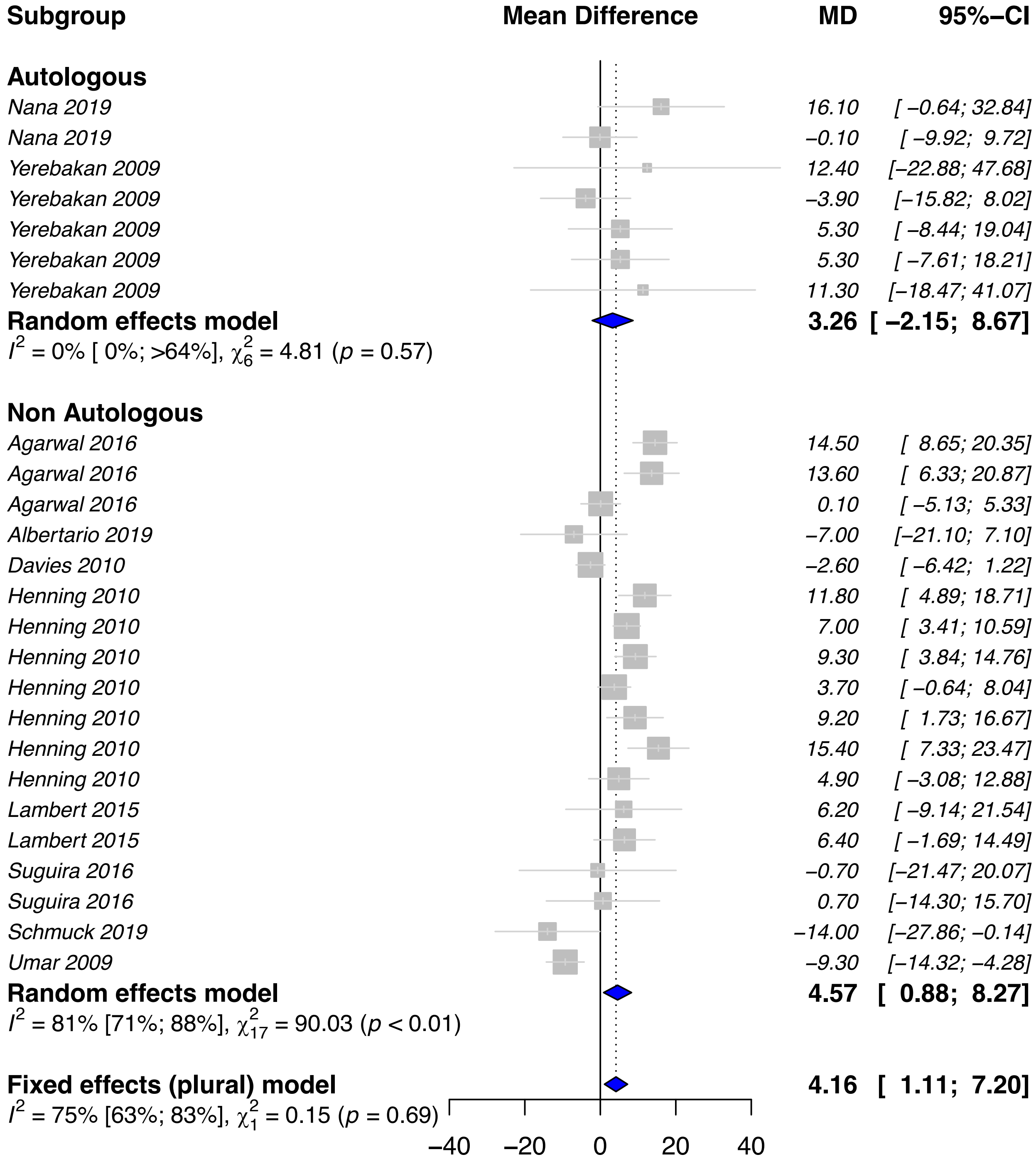

Supplemental 7. E) Cell-based effect on animal ejection fraction by autologous vs. non-autologous sources.

Supplemental 8. Subgroup analysis of regenerative cell effect size on animal fractional shortening. Forest plots demonstrating MD and 95% CI for A) Route of delivery, p=0.001 for intramyocardial. B) Dose, p=0.001 for >10 M cells/kg. C) Tissue Source, p=0 .001 for umbilical cord blood. D) Timing of delivery, p=0.001 for >1 month. E) Disease model, p=0.001 for dilated cardiomyopathy. Cell-based n=175; Control n=171.

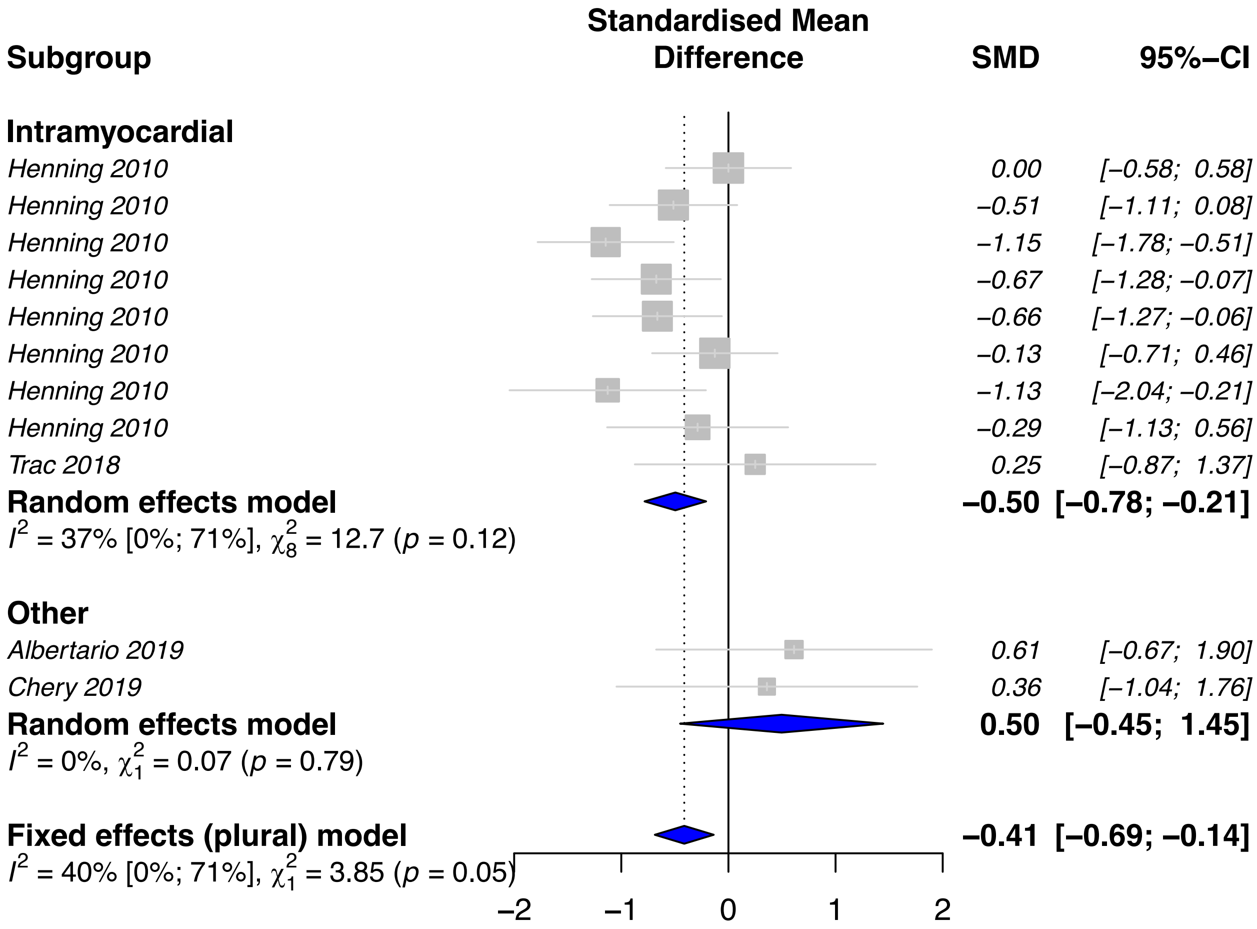

Supplemental 8. A) Cell-based effect on animal FS by route of delivery.

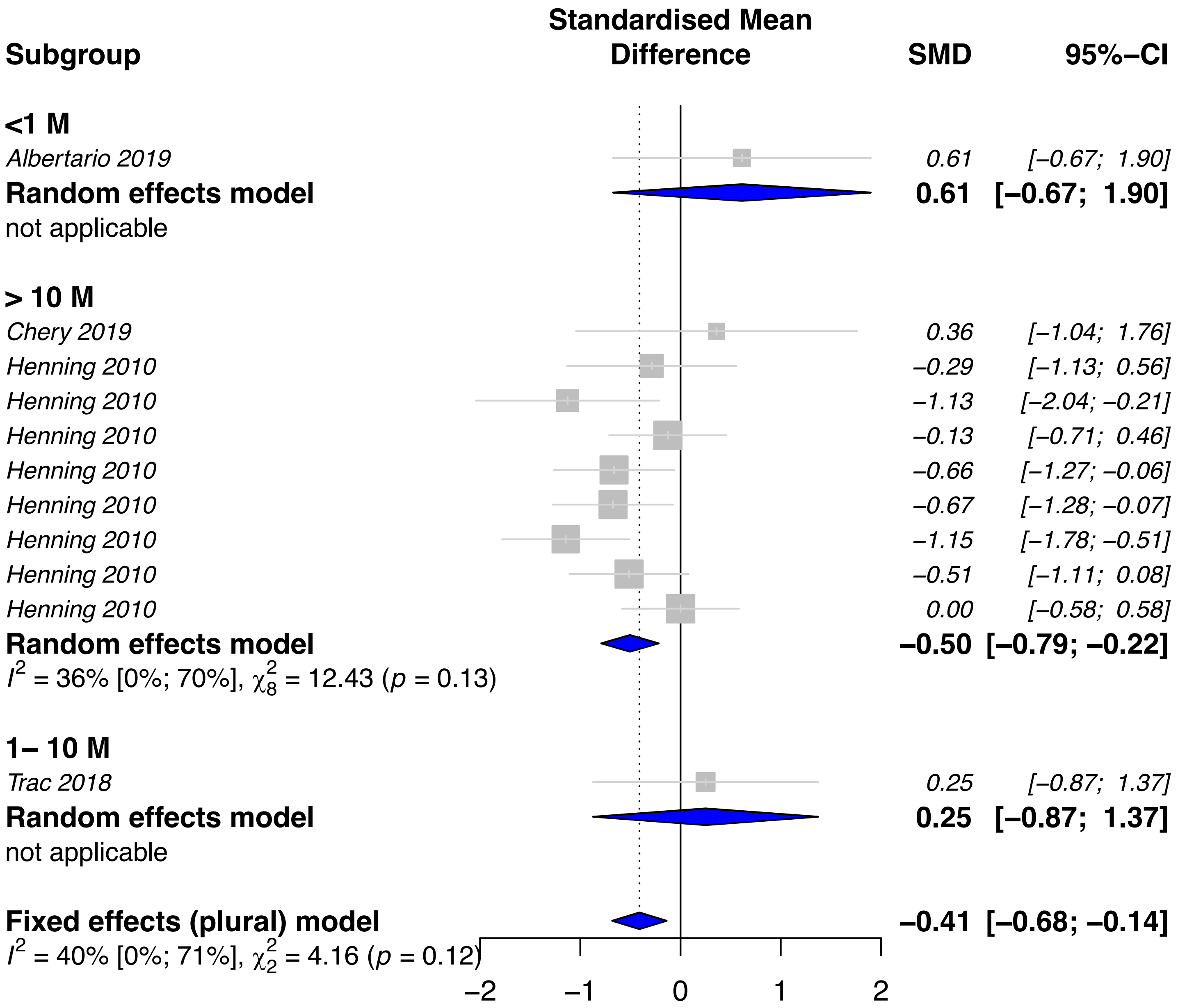

Supplemental 8. B) Cell-based effect on animal FS by dose.

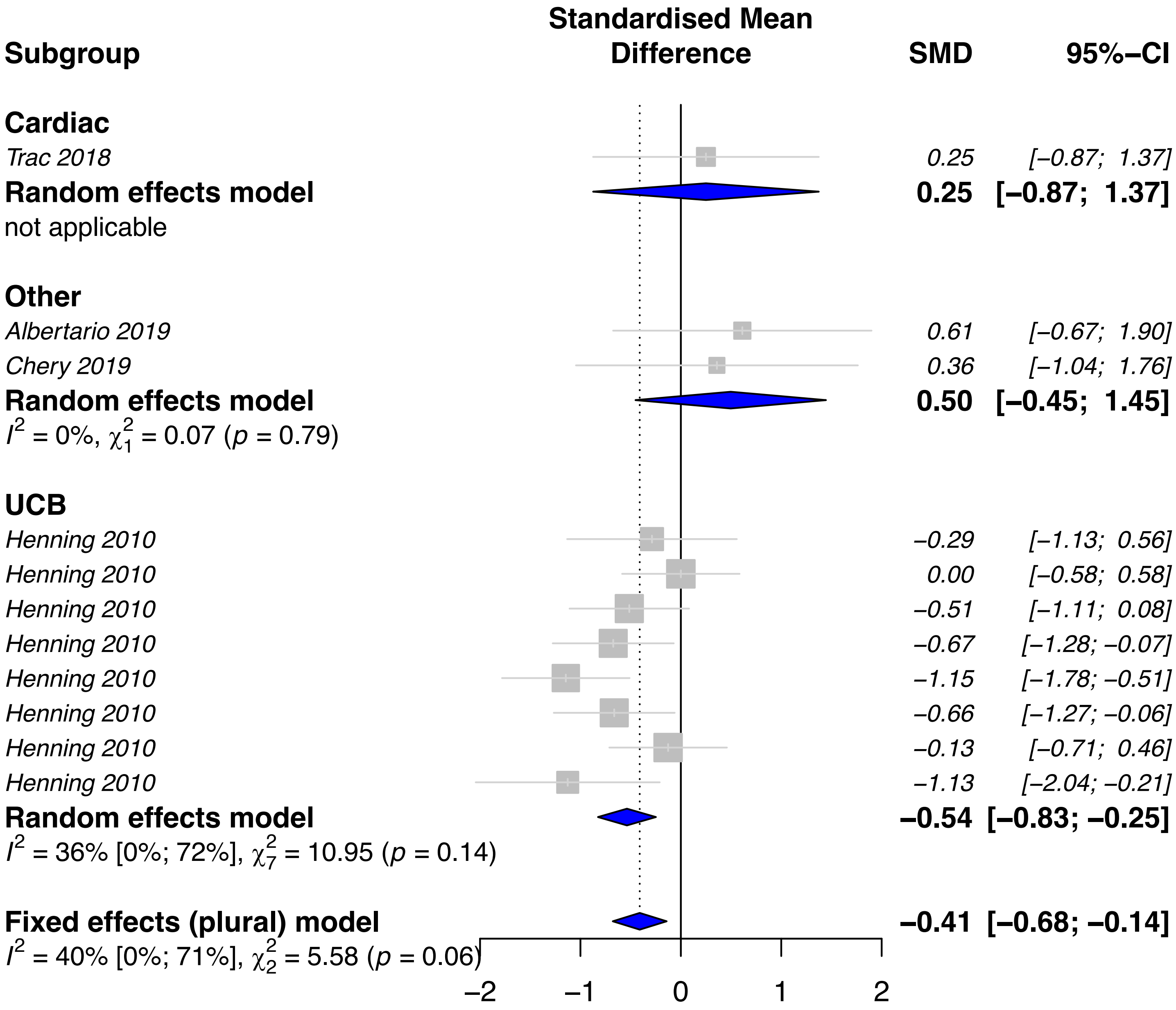

Supplemental 8. C) Cell-based effect on animal FS by tissue source.

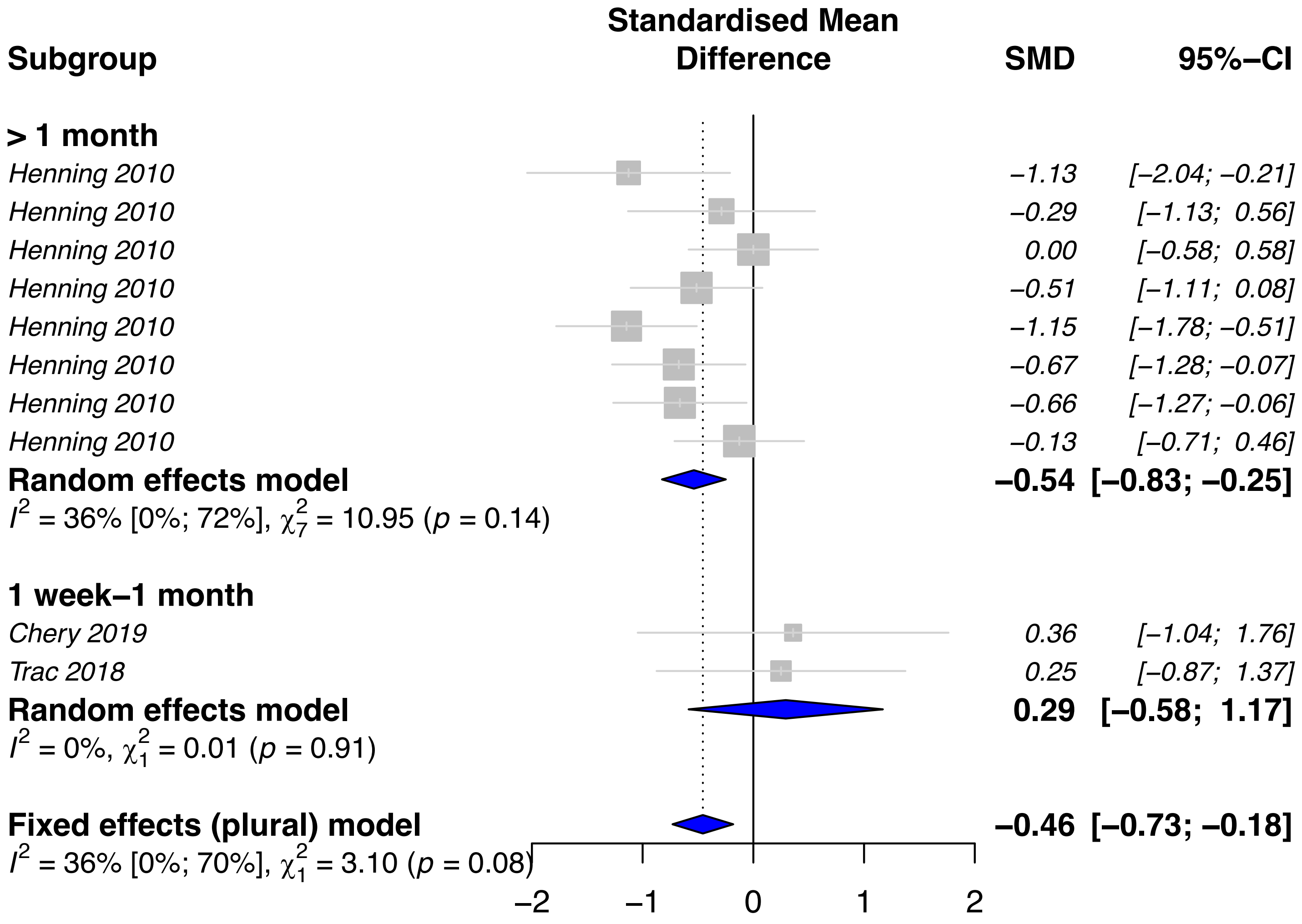

Supplemental 8. D) Cell-based effect on animal FS by timing of delivery.

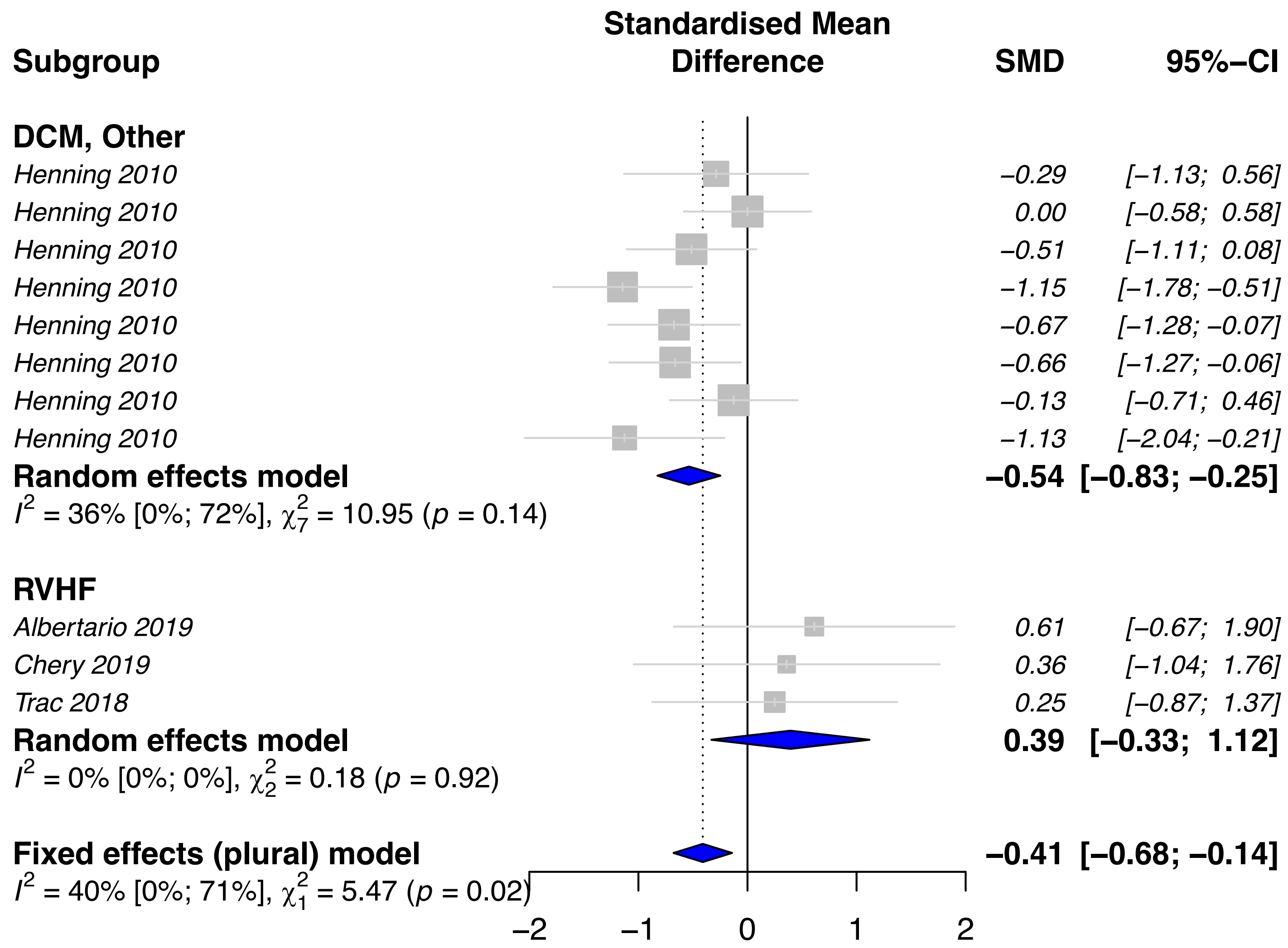

Supplemental 8. E) Cell-based effect on fractional shortening by disease model.

Supplemental 9. Effect size of regenerative cell on additional measures of animal cardiac function. Forest plots demonstrating MD and 95% CI for A) Fractional area change, p=0.05; cell-based n= 33; control n=30. B) End diastolic volume, p=0.48; cell-based n=67; control n=61. C) End systolic volume, p=0.60; cell-based n=72; control n=66. D) Tricuspid annular plane systolic excursion, p=0.55; cell-based n=33; control n=58.

	Experimental			Control					
**Study**	**Total**	**Mean**	**SD**	**Total**	**Mean**	**SD**	**MD**	**95%−CI**	**Weight**
Albertario 2019	5	47.10	7.8000	5	49.20	6.5000	−2.10	[−11.00; 6.80]	17.0%
Trach 2018	8	39.80	14.9000	5	27.30	10.5000	12.50	[−1.33; 26.33]	13.6%
Wehman 2016	5	42.20	6.5000	5	46.00	2.5000	−3.80	[−9.90; 2.30]	18.7%
Wehman 2016	5	47.80	7.6000	5	29.80	10.7000	18.00	[6.50; 29.50]	15.2%
Wehman 2017	5	53.40	6.3000	5	46.00	2.5000	7.40	[1.46; 13.34]	18.8%
Wehman 2017	5	53.20	1.3000	5	29.90	10.5000	23.30	[14.03; 32.57]	16.7%
**Random effects model**	**33**			**30**			**8.65**	**[−0.11; 17.42]**	**100.0%**
**Prediction interval**								**[−21.38; 38.69]**	

Heterogeneity: $I^2 = 84\%$, $\tau^2 = 97.0780$, $p < 0.01$

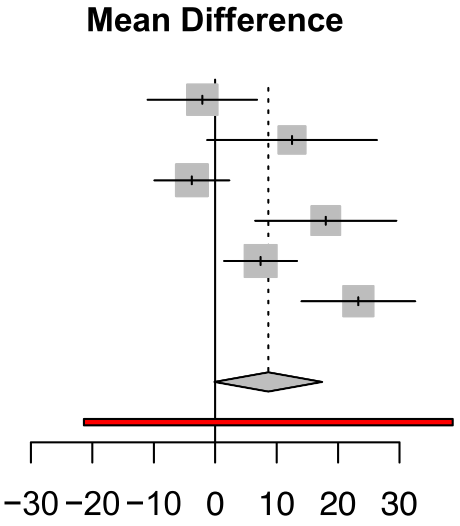

Supplemental 9. A) Cell-based effect on animal FAC.

Study	Experimental Total	Experimental Mean	Experimental SD	Control Total	Control Mean	Control SD	SMD	95%−CI	Weight
Davies 2010	8	27.90	18.70	8	29.40	19.80	−0.07	[−1.05; 0.91]	12.7%
Lambert 2015	6	166.00	112.70	6	148.00	58.80	0.18	[−0.95; 1.32]	9.4%
Lambert 2015	6	192.00	134.70	6	208.00	144.50	−0.11	[−1.24; 1.03]	9.5%
Nana 2019	8	220.00	138.60	6	213.00	193.00	0.04	[−1.02; 1.10]	10.9%
Nana 2019	8	288.00	172.50	6	225.00	154.30	0.36	[−0.71; 1.43]	10.6%
Yerebakan 2009	6	21.60	23.00	6	26.60	17.10	−0.23	[−1.36; 0.91]	9.4%
Yerebakan 2009	9	103.90	32.40	9	90.20	52.80	0.30	[−0.63; 1.23]	14.1%
Yerebakan 2009	8	47.80	20.10	7	49.90	17.70	−0.10	[−1.12; 0.91]	11.8%
Yerebakan 2009	8	27.90	11.00	7	31.10	4.50	−0.35	[−1.37; 0.68]	11.6%
**Random effects model**	**67**			**61**			**0.01**	**[−0.34; 0.36]**	**100.0%**

Heterogeneity: $I^2 = 0\%$, $\tau^2 = 0$, $p = 0.99$

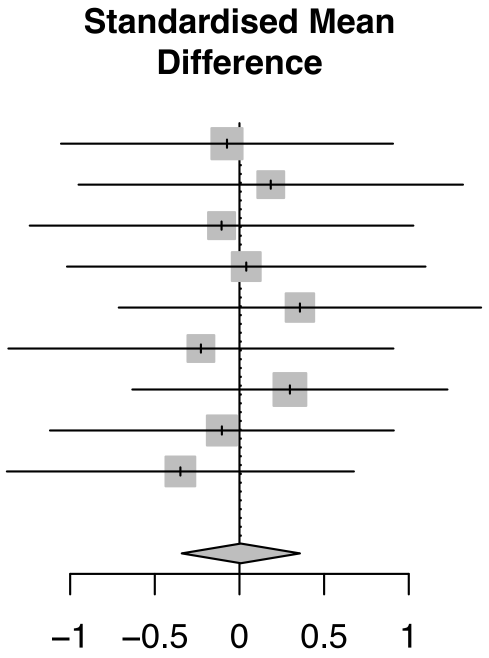

Supplemental 9. B) Cell-based effects on animal EDV.

Study	Experimental Total	Experimental Mean	Experimental SD	Control Total	Control Mean	Control SD	SMD	95%−CI	Weight
Albertario 2019	5	10.00	13.40	5	14.00	2.20	−0.38	[−1.63; 0.88]	7.1%
Davies 2010	8	18.60	10.20	8	17.70	12.70	0.07	[−0.91; 1.05]	11.7%
Lambert 2015	6	67.00	49.00	6	80.00	95.50	−0.16	[−1.29; 0.98]	8.8%
Lambert 2015	6	103.00	88.20	6	83.00	90.60	0.21	[−0.93; 1.34]	8.7%
Nana 2019	8	45.00	39.60	6	47.00	51.40	−0.04	[−1.10; 1.02]	10.0%
Nana 2019	8	100.00	679.00	6	43.00	36.70	0.10	[−0.96; 1.16]	10.0%
Yerebakan 2009	8	19.90	13.60	7	18.20	10.30	0.13	[−0.88; 1.15]	10.9%
Yerebakan 2009	8	9.50	7.90	7	7.80	3.40	0.26	[−0.76; 1.28]	10.8%
Yerebakan 2009	6	9.00	6.40	6	7.20	9.30	0.21	[−0.93; 1.34]	8.7%
Yerebakan 2009	9	43.20	22.20	9	38.90	27.90	0.16	[−0.76; 1.09]	13.1%
**Random effects model**	**72**			**66**			**0.07**	**[−0.26; 0.41]**	**100.0%**

Heterogeneity: $I^2 = 0\%$, $\tau^2 = 0$, $p = 1.00$

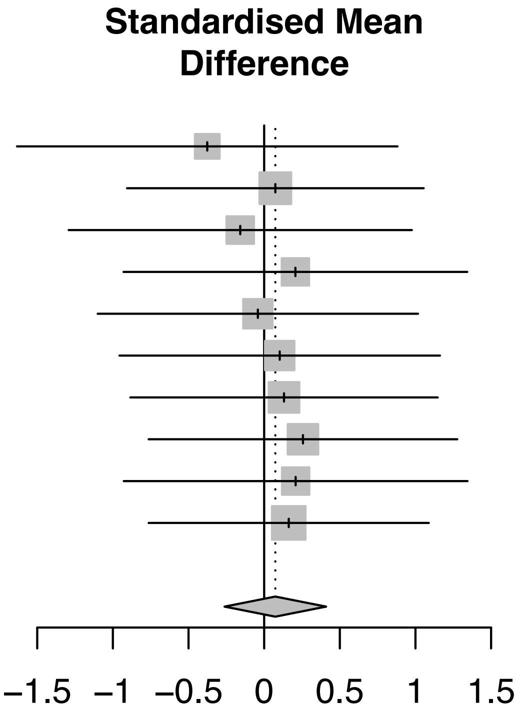

Supplemental 9. C) Cell-based effect on animal ESV.

Study	Experimental Total	Experimental Mean	Experimental SD	Control Total	Control Mean	Control SD	SMD	95%-CI	Weight
Agarwal 2016	4	1.80	0.20	9	1.70	0.30	0.34	[−0.85; 1.52]	13.5%
Agarwal 2016	4	1.60	0.40	9	1.90	0.60	−0.50	[−1.71; 0.70]	13.3%
Trac 2018	5	1.50	0.70	8	0.80	1.40	0.55	[−0.60; 1.69]	14.6%
Trac 2018	5	1.60	1.80	8	1.30	1.10	0.20	[−0.92; 1.32]	15.2%
Trac 2018	5	1.20	1.10	8	1.60	1.10	−0.34	[−1.47; 0.79]	15.0%
Trac 2018	5	1.50	0.50	8	1.80	0.80	−0.40	[−1.53; 0.74]	14.9%
Trac 2018	5	1.50	0.70	8	2.10	0.60	−0.87	[−2.06; 0.31]	13.5%
**Random effects model**	**33**			**58**			**−0.14**	**[−0.58; 0.30]**	**100.0%**

Heterogeneity: $I^2 = 0\%$, $\tau^2 = 0$, $p = 0.61$

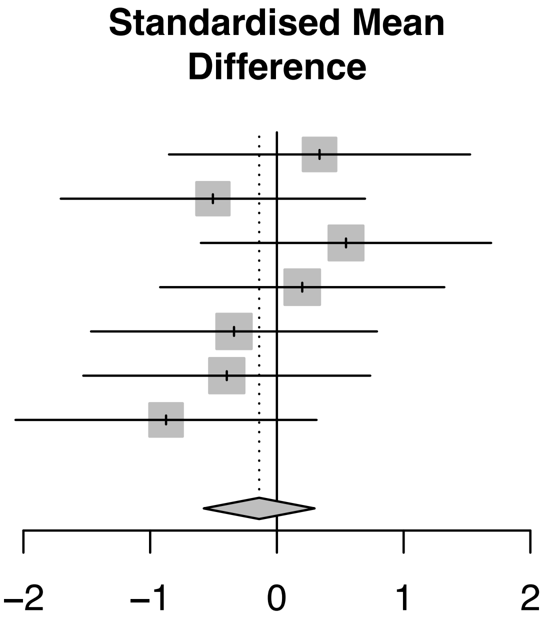

Supplemental 9. D) Cell-based effect on Animal TAPSE.

Supplemental 10. Effect size of regenerative cell on additional measures of human cardiac function. Forest plots demonstrating MD and 95% CI for A) Fractional area change, p-0.19; cell-based n=62; control n=62. B) End diastolic volume, p=0.52; cell-based n=110; control n=110. C) End systolic volume, p=0.96; cell-based n=110; control n=110.

Study	Experimental Total	Experimental Mean	Experimental SD	Control Total	Control Mean	Control SD	MD	95%-CI	Weight
Ishigami 2017	17	33.10	15.30	17	32.50	12.00	0.60	[−8.64; 9.84]	18.8%
Ishigami 2017	17	33.60	16.90	17	34.60	9.10	−1.00	[−10.12; 8.12]	19.3%
Tarui 2015	7	33.60	8.70	7	34.20	11.60	−0.60	[−11.34; 10.14]	13.9%
Tarui 2015	7	34.10	11.40	7	39.40	5.80	−5.30	[−14.78; 4.18]	17.9%
Tarui 2015	7	33.90	11.90	7	39.20	5.80	−5.30	[−15.11; 4.51]	16.7%
Tarui 2015	7	34.40	13.50	7	39.50	5.80	−5.10	[−15.98; 5.78]	13.5%
**Random effects model**	**62**			**62**			**−2.68**	**[−6.69; 1.32]**	**100.0%**

Heterogeneity: $I^2 = 0\%$, $\tau^2 = 0$, $p = 0.91$

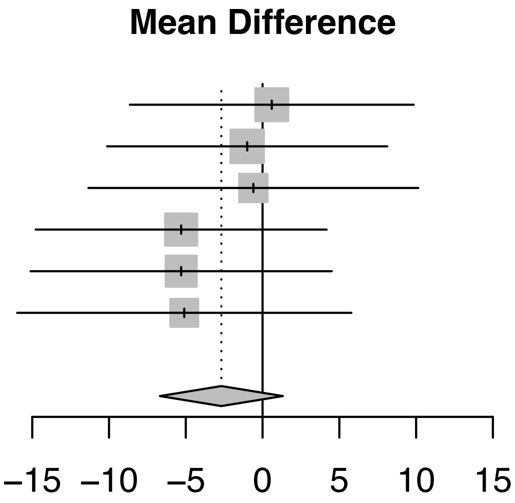

Supplemental 10. A) Cell-based effect on human FAC.

Study	Experimental Total	Experimental Mean	Experimental SD	Control Total	Control Mean	Control SD	MD	95%−CI	Weight
Ishigami 2014	7	119.70	94.20	7	113.20	106.10	6.50	[−98.61; 111.61]	6.9%
Ishigami 2014	7	99.80	65.80	7	104.60	76.20	−4.80	[−79.38; 69.78]	13.8%
Ishigami 2014	7	100.30	55.60	7	94.90	55.30	5.40	[−52.69; 63.49]	22.7%
Ishigami 2017	17	115.80	170.70	17	113.00	113.40	2.80	[−94.62; 100.22]	8.1%
Ishigami 2017	17	100.40	192.50	17	99.50	107.20	0.90	[−103.84; 105.64]	7.0%
Ishigami 2017	17	98.40	169.90	17	95.30	77.10	3.10	[−85.59; 91.79]	9.8%
Ishigami 2017	17	120.90	156.70	17	122.80	149.30	−1.90	[−104.79; 100.99]	7.2%
Tarui 2015	7	160.40	128.60	7	139.00	114.80	21.40	[−106.30; 149.10]	4.7%
Tarui 2015	7	150.20	110.30	7	112.20	83.10	38.00	[−64.30; 140.30]	7.3%
Tarui 2015	7	131.10	69.80	7	101.10	79.90	30.00	[−48.59; 108.59]	12.4%
**Random effects model**	**110**			**110**			**8.99**	**[−18.71; 36.69]**	**100.0%**

Heterogeneity: $I^2 = 0\%$, $\tau^2 = 0$, $p = 1.00$

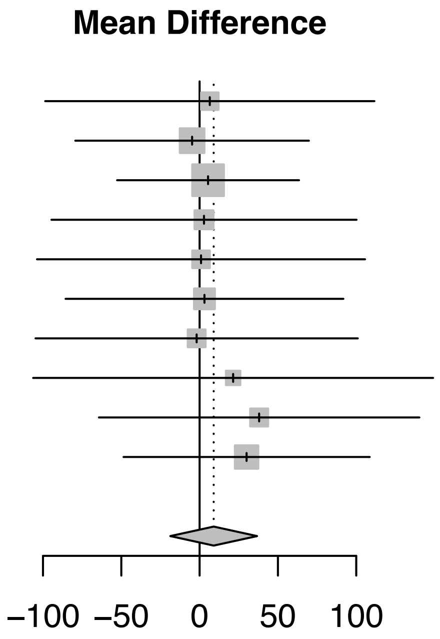

Supplemental 10. B) Cell-based effect on human EDV.

Study	Experimental Total	Experimental Mean	Experimental SD	Control Total	Control Mean	Control SD	MD	95%–CI	Weight
Ishigami 2014	7	60.30	41.30	7	56.90	80.40	3.40	[–63.56; 70.36]	8.3%
Ishigami 2014	7	47.70	35.50	7	41.30	49.20	6.40	[–38.54; 51.34]	18.4%
Ishigami 2014	7	48.50	42.30	7	38.00	33.30	10.50	[–29.38; 50.38]	23.4%
Ishigami 2017	17	82.00	141.00	17	80.90	122.50	1.10	[–87.69; 89.89]	4.7%
Ishigami 2017	17	76.90	140.20	17	66.40	82.50	10.50	[–66.83; 87.83]	6.2%
Ishigami 2017	17	55.90	138.10	17	50.30	80.40	5.60	[–70.36; 81.56]	6.4%
Ishigami 2017	17	51.00	92.40	17	44.00	49.90	7.00	[–42.92; 56.92]	14.9%
Tarui 2015	7	27.40	72.50	7	58.90	49.70	–31.50	[–96.62; 33.62]	8.8%
Tarui 2015	7	34.30	90.70	7	91.60	99.20	–57.30	[–156.87; 42.27]	3.7%
Tarui 2015	7	36.50	96.60	7	67.90	62.40	–31.40	[–116.59; 53.79]	5.1%
**Random effects model**	**110**			**110**			**–0.49**	**[–19.78; 18.79]**	**100.0%**

Heterogeneity: $I^2 = 0\%$, $\tau^2 = 0$, $p = 0.96$

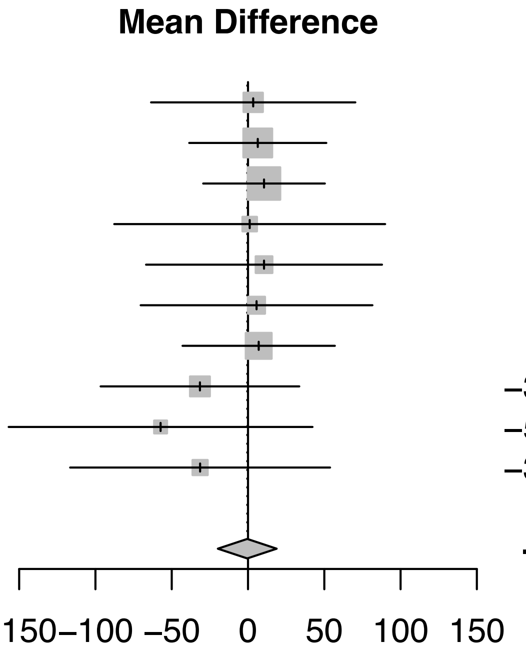

Supplemental 10. C) Cell-based effect on human ESV.

Supplemental 11. SYRCLE risk of bias for animal studies.

Author (Year)	Random sequence generation?	Groups similar at baseline?	Allocation concealed?	Animals randomly housed?	Blinding of caregivers and/or examiners?	Random selection for outcome assessment?	Blinding of outcome assessor?	Incomplete outcome data addressed?	Free from selective outcome reporting?	Free from other bias?
Agarwal (2016)	Yes	Yes	Yes	Unclear	Unclear	No	Yes	Yes	Yes	Yes
Albertario (2019)	Yes	Yes	No	Yes	Unclear	Unclear	Yes	Yes	Yes	Yes
Borenstein (2005)	Yes	Yes	Unclear	Unclear	Unclear	Unclear	Unclear	Yes	Yes	Yes
Brizard (2015)	Yes	Yes	Yes	Yes	Unclear	Unclear	Yes	Yes	Yes	Yes
Cao (2015)	Unclear	Yes	Unclear	Yes	Unclear	Unclear	Yes	Yes	Yes	Yes
Chery (2019)	Unclear	Yes	No	Unclear	Unclear	Unclear	Yes	Yes	Yes	Yes
Davies (2010)	Unclear	Yes	Unclear	Unclear	Unclear	Unclear	Unclear	Yes	Yes	No
Henning (2010)	Yes	Yes	Unclear	Unclear	Unclear	Unclear	Yes	Yes	Yes	Yes
Lambert (2015)	Unclear	Yes	Unclear	Unclear	Unclear	Unclear	Yes	Yes	Yes	Yes
Liu (2011)	Unclear	Yes	Unclear	Yes	Unclear	Unclear	Yes	Yes	Yes	Yes
Nana-Leventaki (2019)	Yes	Yes	Unclear	Yes	Unclear	Unclear	Yes	Yes	Yes	Yes
Schmuck (2019)	Yes	Yes	Unclear	Unclear	Unclear	Unclear	Yes	Yes	Yes	Yes
Sugiura (2016)	Yes	Yes	Unclear	Yes	Unclear	Unclear	Unclear	Yes	Yes	Yes
Trac (2018)	Yes	Yes	Unclear	Yes	Unclear	Unclear	Yes	Yes	Yes	Yes
Umar (2009)	Unclear	Yes	Unclear	Unclear	Unclear	Unclear	Yes	Yes	Yes	Yes
Wehman (2016)	Unclear	Yes	Unclear	Unclear	Unclear	Unclear	Yes	Yes	Yes	Yes
Wehman (2017)	Unclear	Yes	Unclear	Unclear	Unclear	Unclear	Yes	Yes	Yes	Yes
Yerebakan (2009)	Yes	Yes	Unclear	Unclear	Unclear	Unclear	Yes	Yes	Yes	Yes

Supplemental 12. ROBINS-I risk of bias for human studies.

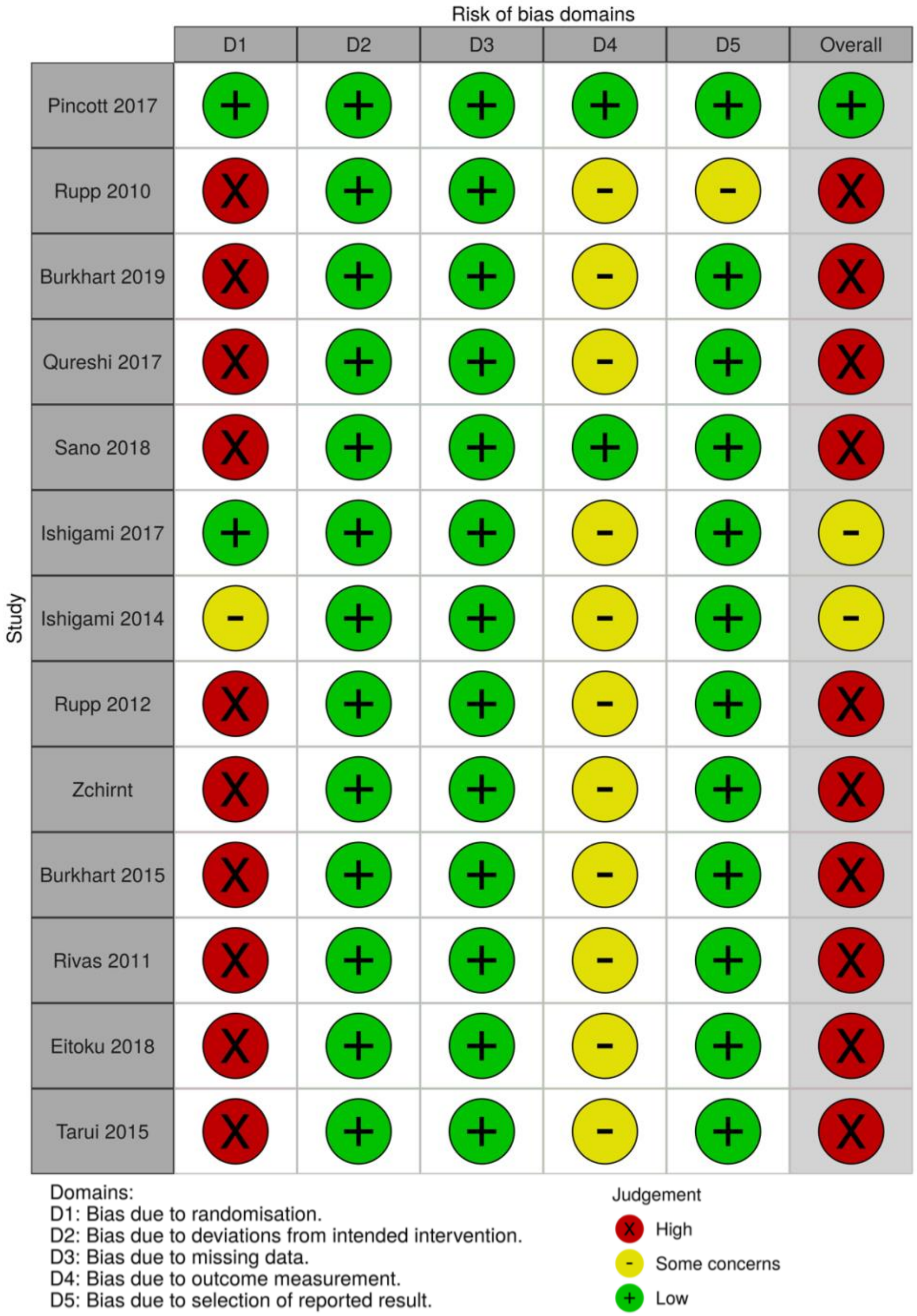

460 **Supplemental 13. Funnel plot diagram for animal ejection fraction.**

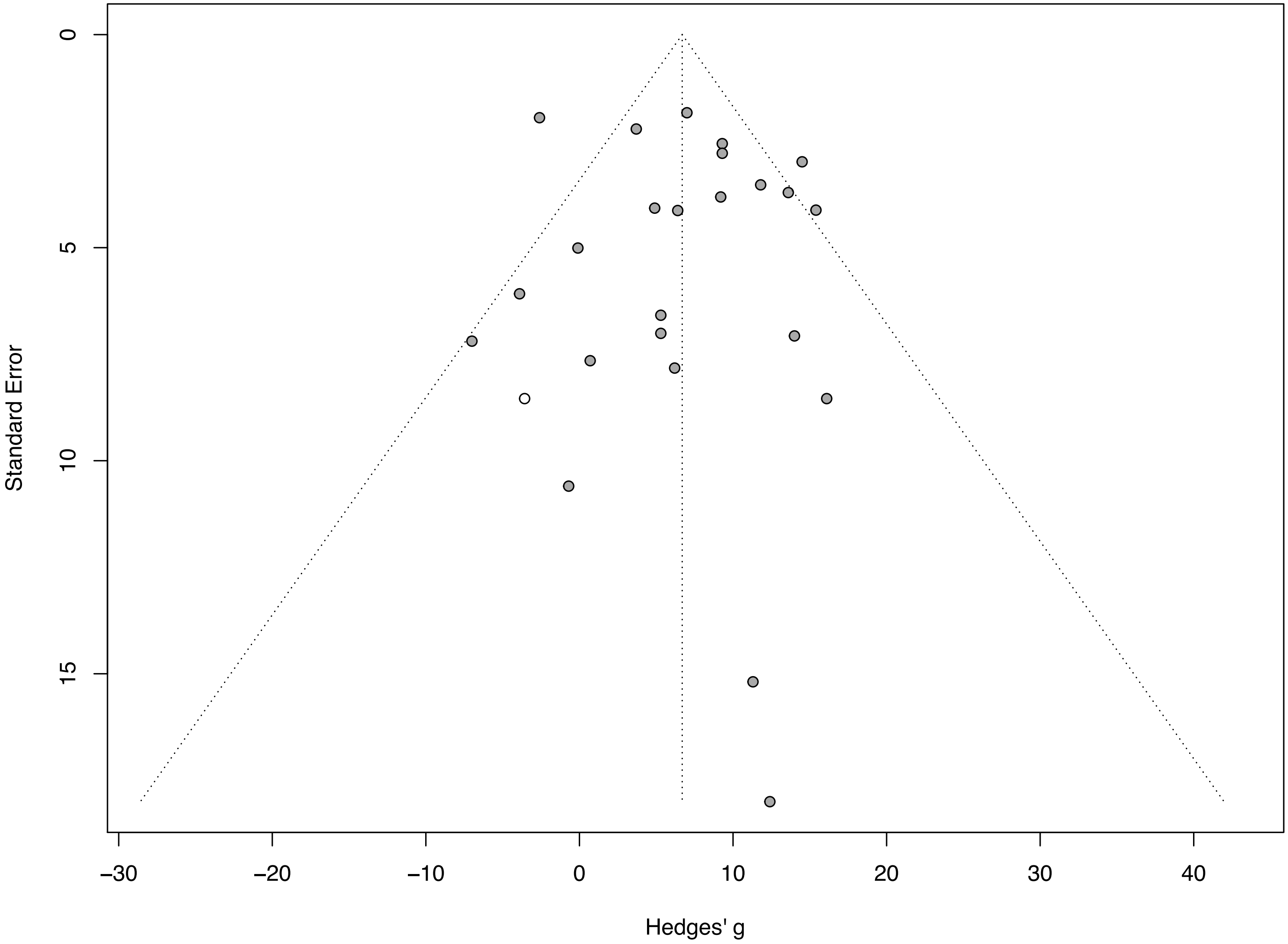

Supplemental 14. Funnel plot diagram for animal fractional shortening.

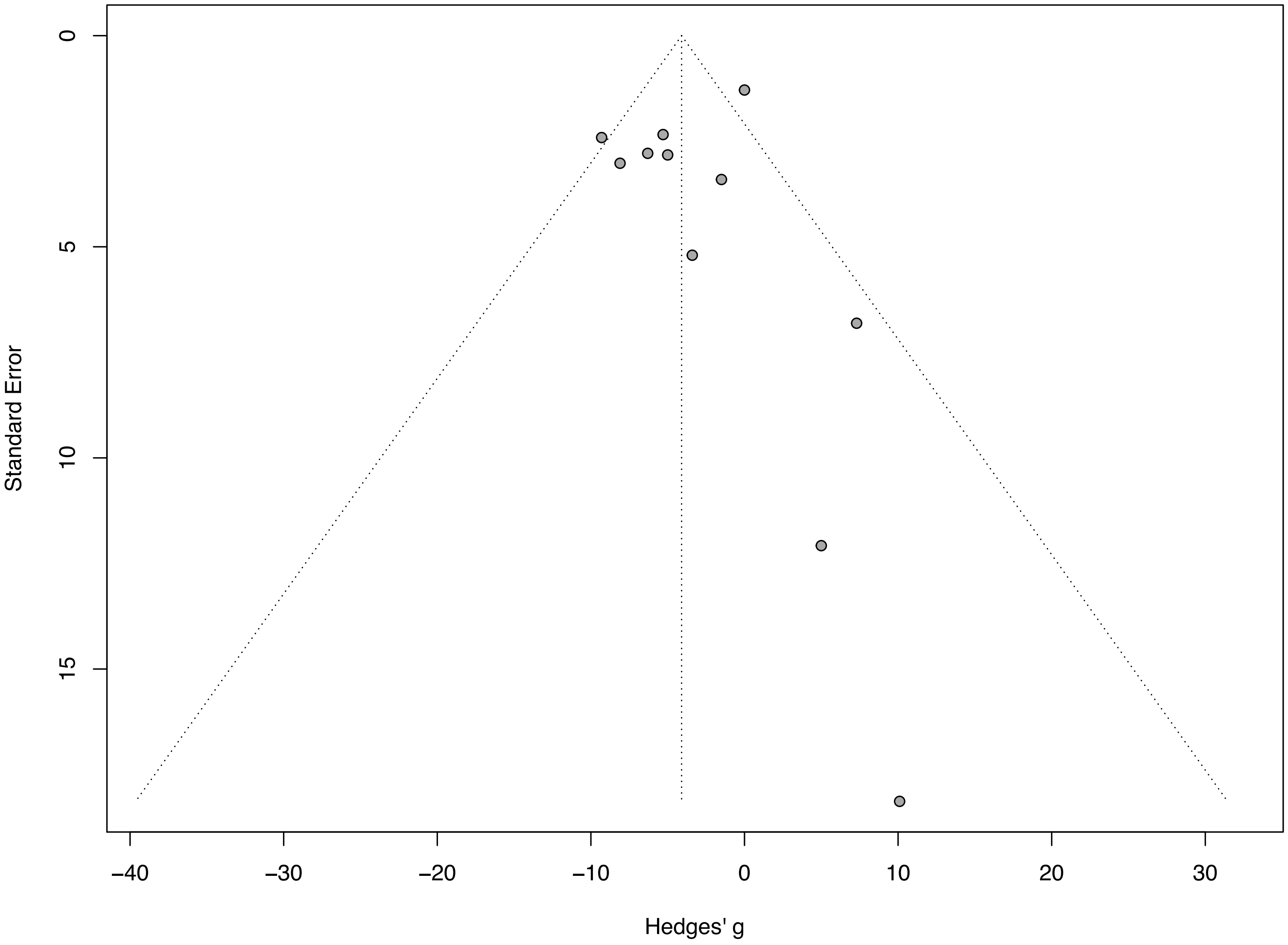

Supplemental Table 1. Animal study intervention characteristics.

Animal Characteristics	n (%)
Disease model	
RVHF	307 (80%)
DCM	45 (12%)
Autoimmune myocarditis	22 (6%)
Infant cardiopulmonary bypass	12 (3%)
Species	
Rats	120 (31%)
Sheep	118 (31%)
Rabbit	50 (13%)
Hamsters	45 (12%)
Swine	42 (11%)
Ram	11 (3%)
Age	
≤ 1 month	143 (37%)
>1 month	128 (33%)
Did Not Report	115 (30%)

Intervention Characteristics	n (%)
Cell route	
Intravenous	140 (36%)
Intramyocardial	135 (35%)
Graft/Patch/Sheet	61 (16%)
Intracoronary	34 (9%)
Epicardial	16 (4%)
Cell dose	
< 1M/kg	109 (28%)
≥ 1M and < 10M/kg	157 (41%)
≥ 10M/kg	120 (31%)
Cell source	
Cardiac	107 (28%)
Umbilical cord blood	93 (24%)
Bone marrow	80 (21%)
Adipose	70 (18%)
Thymus	25 (6%)
Skeletal muscle	11 (3%)
Timing	
Intraoperative and < 24 hours	79 (20%)
≥ 24 hours and < 1 week	0 (0%)
≥ 1 week and < 1 month	100 (26%)
≥ 1 month	177 (46%)
Did Not Report	30 (8%)

Supplemental Table 2. Adverse events by systems.

Acute Adverse Events
Systemic
Allergic reaction / Anaphylaxis
Death
Elevated CRP
Fever
Hemodynamic instability / Hypotension
Malignancy
Tumor formation
Respiratory
Bronchitis
Chest tube
Death - Respiratory
Pleural effusion
Pneumonia
Cardiac
APCA coil occlusion
Arrythmia
Bradycardia
Cardiac tamponade
Cardiac-related pneumothorax
Cardiopulmonary resuscitation
Coronary spasm
Death - Cardiac
ECMO
Epicardial bleed
Heart failure
Myocardial ischemia
Palpitations
Valve malfunction
Neurological
Seizure
Stroke
Hematological
Embolism
Thromboembolic events
GI
Cirrhosis
Protein-losing enteropathy
Renal
Renal deterioration
Infectious disease
Infection

Delayed Adverse Events
Systemic
Late death
Cardiac
Late heart failure

Unplanned Interventions
BCPS or TCPC take down
Catheterizations
Heart transplant
Intubation
Pacemaker implantation

Unplanned Hospitalizations
CCU admission
ED visit
General wards/floor admission
Rehospitalization for heart failure

APCA = aortopulmonary collateral artery
BCPS = bidirectional cavopulmonary shunt
CCU = cardiac/coronary care unit
ED = emergency department
TCPC = total cavopulmonary connection

Supplemental Table 3. Animal adverse events.

Adverse Event	Control		Cell-based		Overall
	Events	Total	Events	Total	Peto OR (95% CI)
Cardiac	13	73	8	74	0.48 (0.17, 1.33)
Respiratory	5	69	8	67	2.29 (0.67, 7.84)
GI	0	69	0	67	-
Hematologic	0	69	0	67	-
Infectious disease	0	69	0	67	-
Systemic	1	69	0	67	0.44 (0.02, 12.01)
Overall	19	418	16	409	0.89 (0.43, 1.83)

Supplemental Table 4. Human study intervention characteristics.

Human Characteristics	n (%)
Disease	
SV	142 (66%)
HLHS	41 (19%)
DCM	32 (15%)
Cell route	
Intracoronary	204 (95%)
Intramyocardial	11 (5%)
Cell dose	
< 1M/kg	170 (79%)
≥ 1M and < 10M/kg	35 (16%)
≥ 10M/kg	0 (0%)
Did Not Report	10 (5%)
Cell source	
Cardiac	170 (79%)
Bone marrow	34 (16%)
Umbilical cord blood	11 (5%)

Supplemental Table 5. Human adverse events.

Adverse Event	Control		Cell-based		Overall
	Events	Total	Events	Total	Peto OR (95% CI)
Cardiac	81	101	41	82	0.11 (0.05, 0.23)
Respiratory	5	101	0	82	0.16 (0.03, 0.95)
GI	2	101	0	82	0.14 (0.01, 2.16)
Hematologic	7	101	2	82	0.44 (0.11, 1.75)
Infectious disease	1	101	0	82	0.19 (0.00, 10.05)
Systemic	2	101	1	82	0.74 (0.07, 7.54)
Overall	98	606	44	492	0.17 (0.09, 0.30)

Supplemental Table 6. Clinical trials, ongoing.

Sponsor	Disease	Methods	Participants	Interventions	Comparison	Outcome	Notes
NCT03779711	HLHS	Phase II treatment given at time of stage II surgical repair	Children <9 months of age with HLHS or HLHS variant with single ventricular dependent CHD having undergone Stage I surgical repair and Stage II surgical repair	Biologic: Autologous UCB derived mononuclear cells<br><br>Procedure: Stage II surgical repair	Stage II surgical repair alone	Efficacy	Recruiting
NCT03525418	HLHS	Phase I/II: First treatment given at time of stage II surgical repair<br><br>Second treatment given at time of stage II surgical repair vs. placebo	HLHS (all types) requiring stage II surgical intervention	Biologic: Bone marrow derived MSCs<br><br>Procedure: Stage II surgical repair	Stage II surgical repair alone	Safety and Efficacy	Recruiting
NCT03431480	HLHS	Phase I open label safety study	Male and females with antenatally diagnosed HLHS (all types requiring Norwood operation)	Biologic: Autologous human placental cord blood mononuclear cells<br><br>Procedure: Stage I surgical repair	N/A	Safety and Efficacy	Recruiting
NCT03079401	HLHS<br><br>AV Canal Defects	Phase I/II	Patient with a history of single ventricle palliation undergoing bidirectional Glenn with LV recruitment procedures or those patients undergoing LV recruitment procedures	Biologic: Mesenchymal progenitor cells	Surgical repair alone	Safety and Efficacy	Recruiting
NCT02781922	HLHS<br><br>SV	Phase III single blind parallel group study	Functional single ventricle patient with HF scheduled for stage 2 (Glenn) or stage 3 (Fontan) surgery	Biologic: Autologous cardiac stem cells	Surgical repair alone	Safety and Efficacy	Unknown

			EF < 55%				
NCT01883076	HLHS	Phase I	Individuals with HLHS who have undergone Stage I surgical palliation and undergoing planned Stage II Palliative Glenn Surgery	Biologic: autologous umbilical cord blood cells	N/A	Safety	Recruiting
